# *Shank3* Exons 14–16 Deletion in Glutamatergic Neurons Leads to Social and Repetitive Behavioral Deficits Associated With Increased Cortical Layer 2/3 Neuronal Excitability

**DOI:** 10.3389/fncel.2019.00458

**Published:** 2019-10-10

**Authors:** Taesun Yoo, Heejin Cho, Haram Park, Jiseok Lee, Eunjoon Kim

**Affiliations:** ^1^Department of Biological Sciences, Korea Advanced Institute of Science and Technology (KAIST), Daejeon, South Korea; ^2^Center for Synaptic Brain Dysfunctions, Institute for Basic Science (IBS), Daejeon, South Korea

**Keywords:** autism, Phelan-McDermid syndrome, Shank3, medial prefrontal cortex, striatum, social interaction, repetitive behavior

## Abstract

Shank3, an abundant excitatory postsynaptic scaffolding protein, has been associated with multiple brain disorders, including autism spectrum disorders (ASD) and Phelan-McDermid syndrome (PMS). However, how cell type-specific *Shank3* deletion affects disease-related neuronal and brain functions remains largely unclear. Here, we investigated the impacts of *Shank3* deletion in glutamatergic neurons on synaptic and behavioral phenotypes in mice and compared results with those previously obtained from mice with global *Shank3* mutation and GABAergic neuron-specific *Shank3* mutation. Neuronal excitability was abnormally increased in layer 2/3 pyramidal neurons in the medial prefrontal cortex (mPFC) in mice with a glutamatergic *Shank3* deletion, similar to results obtained in mice with a global *Shank3* deletion. In addition, excitatory synaptic transmission was abnormally increased in layer 2/3 neurons in mice with a global, but not a glutamatergic, *Shank3* deletion, suggesting that Shank3 in glutamatergic neurons are important for the increased neuronal excitability, but not for the increased excitatory synaptic transmission. Neither excitatory nor inhibitory synaptic transmission was altered in the dorsal striatum of *Shank3*-deficient glutamatergic neurons, a finding that contrasts with the decreased excitatory synaptic transmission in global and *Shank3*-deficient GABAergic neurons. Behaviorally, glutamatergic *Shank3*-deficient mice displayed abnormally increased direct social interaction and repetitive self-grooming, similar to global and GABAergic *Shank3*-deficient mice. These results suggest that glutamatergic and GABAergic *Shank3* deletions lead to distinct synaptic and neuronal changes in cortical layer 2/3 and dorsal striatal neurons, but cause similar social and repetitive behavioral abnormalities likely through distinct mechanisms.

## Introduction

Shank3 is an abundant scaffolding protein mainly present in the postsynaptic side of excitatory synapses and contributes to excitatory synapse development and function (Boeckers et al., 2002; Sheng and Hoogenraad, 2007; Sala et al., 2015). Mutations in the *SHANK3* gene have been associated with various brain disorders, including autism spectrum disorders (ASD), Phelan-McDermid Syndrome (PMS), schizophrenia, intellectual disability, and mania (Wilson et al., 2003; Gauthier et al., 2010; Bonaglia et al., 2011; Hamdan et al., 2011; Phelan and McDermid, 2012; Boccuto et al., 2013; Han et al., 2013; Guilmatre et al., 2014; Leblond et al., 2014; Cochoy et al., 2015). Mechanisms underlying the development of Shank3-related brain dysfunctions have been suggested through studies of a large number of *Shank3*-mutant mice (Bozdagi et al., 2010; Peca et al., 2011; Wang X. et al., 2011; Yang et al., 2012; Han et al., 2013; Jiang and Ehlers, 2013; Kouser et al., 2013; Duffney et al., 2015; Lee et al., 2015; Sala et al., 2015; Speed et al., 2015; Jaramillo et al., 2016, 2017; Mei et al., 2016; Wang et al., 2016, 2019a,b; Zhou et al., 2016; Monteiro and Feng, 2017; Vicidomini et al., 2017; Bey et al., 2018; Drapeau et al., 2018; Qin et al., 2018; Yoo et al., 2018).

More recently, *Shank3* deletions restricted to specific brain regions and cell types in mice have been attempted (Bey et al., 2018; Yoo et al., 2018). For instance, *Shank3* (exons 4–22) deletions in glutamatergic and striatal D1 and D2 GABAergic neurons have been generated using cell type-specific Cre mouse lines, including NEX (dorsal telencephalic glutamatergic neurons mainly found in the cortex and hippocampus), Dlx5/6 (striatal GABAergic neurons), and Drd1/2 (dopaminergic D1 or D2 neurons) (Bey et al., 2018). In addition, a *Shank3* (exons 14–16) deletion restricted to GABAergic neurons has also been developed (Yoo et al., 2018). These different *Shank3* deletions result in shared as well as distinct phenotypes, including striatal synaptic dysfunctions and ASD-related behavioral deficits (Bey et al., 2018; Yoo et al., 2018).

We previously reported a GABAergic *Shank3* (exons 14–16) deletion and described its impacts on synaptic and behavioral phenotypes in mice (Yoo et al., 2018); however, *Shank3* is expressed in both glutamatergic and GABAergic neurons (Han et al., 2013; Yoo et al., 2018). In addition, although a glutamatergic *Shank3* (exons 4–22) deletion has previously been generated using the NEX-Cre driver line (Bey et al., 2018), in our conditional knockout (cKO)-ready *Shank3*-mutant mice, exons 14–16 rather than exons 4–22 were targeted, affecting different Shank3 splice variants and leading to different phenotypes, based on the complex alternative splicing patterns in the *Shank3* gene (Lim et al., 2001; Durand et al., 2007; Wang X. et al., 2011; Jiang and Ehlers, 2013; Wang et al., 2014a, b; Monteiro and Feng, 2017). We thus attempted to use *Emx1*-Cre that drives gene expression mainly in the cortex and hippocampus derived from the dorsal telencephalon (Gorski et al., 2002).

The striatum has been strongly associated with ASD (Haznedar et al., 2006; Di Martino et al., 2011; Langen et al., 2013; Kohls et al., 2014; Rothwell et al., 2014; Barak and Feng, 2016; Fuccillo, 2016; Schuetze et al., 2016; Rapanelli et al., 2017). In addition, many previous studies have identified dysfunctions in the corticostriatal pathway involving the dorsal striatum in *Shank3*-mutant mice (Peca et al., 2011; Filice et al., 2016; Fuccillo, 2016; Jaramillo et al., 2016, 2017; Mei et al., 2016; Peixoto et al., 2016; Wang et al., 2016, 2017; Zhou et al., 2016; Lee Y. et al., 2017; Reim et al., 2017; Vicidomini et al., 2017; Bey et al., 2018; Fourie et al., 2018; Yoo et al., 2018). The medial prefrontal cortex (mPFC) has also been strongly implicated in ASD (Ernst et al., 1997; Mundy, 2003; Pierce et al., 2004; Carper and Courchesne, 2005; Amodio and Frith, 2006; Gilbert et al., 2008; Rinaldi et al., 2008; Shalom, 2009; Courchesne et al., 2011; Yizhar et al., 2011; Testa-Silva et al., 2012; Liang et al., 2015; Barak and Feng, 2016; Ko, 2017; Wang et al., 2019c). However, how the striatum and prefrontal cortex differentially contribute to core ASD-related behaviors remains unclear.

Different cortical layers such as layer 2/3 and layer 5 have been suggested to contribute to ASD. Some previous studies have characterized layer 5 cortical pyramidal neurons in the mPFC or somatosensory cortex in *Shank3*-mutant mice (Peixoto et al., 2016; Qin et al., 2019; Wang et al., 2019d). Layer 2/3 pyramidal neurons also receive diverse inputs from intracortical and subcortical afferents and provide excitatory inputs onto other cortical layers, including layer 5 (Gabbott et al., 2005; Hoover and Vertes, 2007; Xu and Sudhof, 2013; Lee et al., 2014; Virtanen et al., 2018), and have been implicated in cortical neuronal integration, cognitive functions, and brain disorders such as ASD, schizophrenia, and depression (Parikshak et al., 2013; Shrestha et al., 2015; Li et al., 2016; Page et al., 2018). Indeed, previous studies have highlighted the importance of layer 2/3 cortical neurons in ASD, reporting that superficial cortical layers in the human brain are enriched for genes that are coexpressed in ASD (Parikshak et al., 2013), that inhibitory synaptic transmission in layer 2/3 mPFC cortical neurons is reduced in neuroligin-2–mutant mice with cognitive and social dysfunctions (Liang et al., 2015), and that enhanced synapse remodeling in layer 2/3 pyramidal neurons may be a common pathology in two independent mouse models of ASD (Isshiki et al., 2014). A more recent study reported age-dependent changes in excitatory synaptic transmission and spine density in layer 2/3 mPFC pyramidal neurons (Zhou et al., 2016). However, the role of layer 2/3 pyramidal neurons in ASD-related brain dysfunctions remains incompletely studied.

In the present study, we generated a *Shank3* (exons 14–16) deletion in mice restricted to glutamatergic neurons and investigated its impact on mPFC layer 2/3 and dorsal striatal neurons and ASD-related behaviors, and compared the results with those obtained in mice with global or GABAergic *Shank3* deletions. Our findings indicate that layer 2/3 pyramidal neurons from *Emx1-Cre;Shank3^*fl/fl*^* (*Emx1-Cre;Shank3^Δ14–16^*) mice showed increased neuronal excitability, similar to results in global *Shank3*-deficient (global *Shank3^Δ14–16^*) layer 2/3 neurons. In contrast to the decreased excitatory synaptic transmission in global *Shank3^Δ14–16^* and GABAergic *Shank3*-deficient (*Viaat-Cre;Shank3^Δ14–16^*) neurons, *Emx1-Cre;Shank3^Δ14–16^* dorsolateral striatal neurons showed normal synaptic transmission. Behaviorally, *Emx1-Cre;Shank3^Δ14–16^* mice showed abnormal social interaction and self-grooming, similar to global *Shank3^Δ14–16^* and *Viaat-Cre;Shank3^Δ14–16^* mice. These results suggest that both glutamatergic and GABAergic *Shank3* deletions contribute to the social and repetitive behavioral deficits observed in global *Shank3^Δ14–16^* mice, but lead to distinct synaptic and neuronal alterations in layer 2/3 pyramidal neurons and dorsal striatal GABAergic neurons.

## Materials and Methods

### Animals

Mice carrying a deletion of exons 14–16 of the *Shank3* gene flanked by LoxP sites have been described (Yoo et al., 2018). Homozygous *Shank3*^Δ14–16^ cKO mice with a gene deletion restricted to dorsal telencephalic excitatory neurons (*Emx1-Cre;Shank3^*fl/fl*^* mice) were produced by crossing homozygous *Shank3*^*fl/fl*^ female mice with double-heterozygous *Emx1-Cre;Shank3^*fl/*^*^+^ mice. Cre-negative *Shank3*^*fl/fl*^ littermates, referred to as wild-type (WT) throughout the manuscript, were used as controls for *Emx1-Cre;Shank3^*fl/fl*^* mice. The *Emx1-Cre* mouse line, purchased from the Jackson Laboratory (Jackson; #005628) and maintained in a C57BL/6J genetic background for more than five generations, was used for comparisons with all global and cKO mouse lines in the same pure C57BL/6J background. Mice were bred and maintained at the mouse facility of Korea Advanced Institute of Science and Technology (KAIST) according to Animal Research Requirements of KAIST, and all procedures were approved by the Committee of Animal Research at KAIST (KA2016-30). Animals were fed *ad libitum* and housed under a 12-h light/dark cycle (light phase from 1:00 am to 1:00 pm). Genotypes of *Emx1-Cre;Shank3^*fl/fl*^* mice were determined by polymerase chain reaction (PCR) using the following primer pairs: floxed (478 bp) or WT allele (276 bp), 5′-GGG TTC CTA TGA CAG CCT CA-3′ (forward) and 5′-TTC TGC AGG ATA GCC ACC TT-3′ (reverse); Emx1-Cre (272 bp), 5′-GTG TTG CCG CGC CAT CTG C-3′ (forward) and 5′-CAC CAT TGC CC TGT TTC ACT ATC-3′ (reverse). Only male mice were used for behavioral and electrophysiological experiments, whereas both male and female were used for biochemical experiments.

### Brain Lysates

Brains from *Emx1-Cre; Shank3^*fl/fl*^* mice and their *Shank3*^*fl/fl*^ littermates (12 weeks; females for Shank3 protein levels, 13 weeks; males for Shank1 and Shank2 protein levels) were extracted and dissected on ice into cortex, thalamus, striatum, and hippocampus, followed by homogenization with ice-cold homogenization buffer (0.32 M sucrose, 10 mM HEPES, pH 7.4, 2 mM EDTA, pH 8.0, 2 mM EGTA, pH 8.0, protease inhibitors, phosphatase inhibitors). Total lysates were prepared by boiling with β-mercaptoethanol directly after homogenization.

### Western Blot

Total brain lysates separated in electrophoresis and transferred to a nitrocellulose membrane were incubated with primary antibodies to Shank1 (#2100, guinea pig) (Ha et al., 2016), Shank2 (Synaptic Systems 162 202), Shank3 (#2036 guinea pig polyclonal antibodies raised against aa 1289–1318 of the mouse Shank3 protein) (Lee et al., 2015) and α-tubulin (Sigma T5168) at 4°C overnight. Fluorescent secondary antibody signals were detected using Odyssey^®^ Fc Dual Mode Imaging System.

### Behavioral Assays

Male mice (2–8-mo-old) were used for all behavioral assays. Before behavioral experiments, mice were handled for 10 min per day for 3 days. All behavioral assays were initiated after a 30-min habituation in a dark booth. The behavioral tests for *Emx1-Cre;Shank3^Δ14–16^* mice and *Emx1-Cre* mice were performed in the order indicated in Supplementary Table S1. The order of behavioral tests was designed to minimize stress to the animals.

### Three-Chamber Test

Social approach and social novelty recognition were measured using the three-chambered social interaction test (Crawley, 2004; Nadler et al., 2004; Moy et al., 2009; Silverman et al., 2010) under illuminated (70–80 lux) conditions. The 3-chamber test apparatus is a white acrylic box (60 × 40 × 20 cm) divided into three chambers. Both left and right side chambers contained a cage in the upper or lower corner for an object or a stranger mouse. Experimental mice were isolated in a single cage for 3 days prior to the test, whereas unfamiliar stranger mice (129S1/SvlmJ strain) were group-housed (5–7 mice/cage). All stranger mice were age-matched males and were habituated to a corner cage during the previous day (30 min). The test consisted of three phases: empty-empty (habituation), stranger 1-object (S1-O), and stranger 1-stranger 2 (S1-S2). In the first phase (habituation), a test mouse was placed in the center area of the three-chambered apparatus, and allowed to freely explore the whole apparatus for 10 min. The mouse was then gently guided to the center chamber while an inanimate blue cylindrical object (O) and a WT stranger mouse (S1) were placed in the two corner cages. The positions of object (O) and stranger 1 (S1) were alternated between tests to prevent side preference. In the S1-O phase, the test mouse was allowed to explore the stranger mouse or the object freely for 10 min. Before the third phase (S1-S2), the subject mouse was again gently guided to the center chamber while the object was replaced with a new WT stranger mouse (S2). The subject mouse again was allowed to freely explore all three chambers and interact with both stranger mice for 10 min. The duration of sniffing, defined as positioning of the nose of the test mouse within 2.5 cm of a cage, was measured using EthoVision XT10 (Noldus) software.

### Direct Social Interaction Test

Direct social interaction tests were performed as described previously (Chung et al., 2015). All mice were isolated for 3 days prior to the day of the experiment. Each individual mouse was habituated to a gray box (30 × 30 × 30 cm; ∼25–30 lux) for two consecutive days (10 min/d). On day 3, pairs of mice of the same genotype (originally housed separately) were placed in the test box for 10 min. Time spent in nose-to-nose interaction, following, and total interaction were measured manually in a blinded manner. Nose-to-nose interaction was defined as sniffing the head part of the other mouse. Following included regular following as well as nose-to-tail sniffing. Total interaction included nose-to-nose interaction, following, body contact, allo-grooming, and mounting.

### Tube Test

The tube test assay was performed as described previously (Wang F. et al., 2011). Mice were group-housed (4 in a cage) for 2 weeks before behavioral experiments. We used transparent acryl tubes with 30-cm length and 3-cm inner diameter. During two-day training sessions, each mouse was trained to pass through the tube in either direction for eight times under illuminated (∼30 lux) conditions. As the mice hesitated to move, they were gently pushed by a plastic bar. After this, 3 days of test sessions were proceeded. Animals went through three more training trials before the test. For the test, two different mice were placed into the opposite ends of the test tube and carefully released to meet in the middle of the tube. The mouse that first retreated from the tube was marked as a “loser.” Among six possible pairs between four cage-mates, two pairs were tested per day. Each mouse was ordered by its rank from 1 to 4.

### Courtship Ultrasonic Vocalization

Adult subject male mice were isolated in their home cage for 3 days before the test, whereas age-matched intruder female mice were group-housed (6–7 mice/cage). We did not measure female cycles on the assumption that group housing might synchronize cycles. Basal ultrasonic vocalizations (USVs) of an isolated male mouse in its home cage under light conditions of ∼60 lux in a soundproof chamber were recorded for 5 min in the absence of a female intruder. Next, a randomly chosen stranger C57BL/6J female mouse was introduced into the cage, and female-induced courtship USVs were recorded for 5 min during free interaction between the male and female. Avisoft SASLab Pro software was used to automatically analyze the number of USV calls, latency to first call, and total duration of calls from recorded USV files. Signals were filtered from 1 to 100 kHz and digitized with a sampling frequency of 250 kHz, 16 bits per sample (Avisoft UltraSoundGate 116H). Spectrograms were generated using the following parameters: FFT length, 256; frame size, 100; window, FlatTop; overlap, 75%. These parameters yielded a frequency resolution of 977 Hz and a temporal resolution of 0.256 ms. Frequencies lower than 25 kHz were filtered out to reduce white background noise.

### Repetitive Behavior and Self-Grooming Test

Each mouse was placed in a lighted (∼60–70 lux), fresh home cage with bedding and recorded for 20 min. Time spent in self-grooming and digging behavior, measured manually, was determined by analyzing the last 10 min. Self-grooming behavior was defined as stroking or scratching of the body or face, or licking body parts. Digging was defined as scattering bedding using the head and forelimbs. Self-grooming behavior was further analyzed by placing mice in an empty home cage without bedding and recording them for 20 min. Time spent in self-grooming behavior was counted manually in a blinded manner during the last 10 min.

### Laboras Test

Each mouse was placed in a single cage and recorded for 96 consecutive hours from the start of the night cycle. The illumination condition during light-on periods was ∼60 lux. Basal activities (locomotion, climbing, rearing, grooming, eating, and drinking) were recorded and automatically analyzed using the Laboratory Animal Behavior Observation Registration and Analysis System (LABORAS, Metris). Laboras results were not validated by our own manual analyses, given the availability of previous validation results (Van de Weerd et al., 2001; Quinn et al., 2003, 2006; Dere et al., 2015). Mouse movements during the entire 4-day period were used for quantification of behaviors, except for repetitive behavior, for which analyses were restricted to movements during light-off periods, which yielded clearer results.

### Open-Field Test

Mice were placed in the center of an illuminated (90–100 lux) white acrylic box (40 × 40 × 40 cm), and their locomotion was recorded with a video camera for 1 h. The recorded video was analyzed using EthoVision XT10 software (Noldus). The center zone was defined as a 4 × 4-square area at the center of the entire 6 × 6-square region.

### Elevated Plus-Maze

The maze consists of two open arms (30 × 6 cm, ∼180 lux) and two closed arms (30 × 6 cm, ∼20 lux) elevated 75 cm from the floor. Mice were introduced into the center of the apparatus with their head oriented toward the open arms and were allowed to freely explore the environment for 8 min. Amounts of time spent in open or closed arms and number of transitions were measured using EthoVision XT10 software (Noldus).

### Light-Dark Test

The light-dark (LD) apparatus was divided into light (700 lux; 21 × 29 × 20 cm) and dark (∼5 lux; 21 × 13 × 20 cm) chambers separated by an entrance in the middle wall (5 × 8 cm). Mice were introduced into the light chamber with their head oriented toward the opposite side of the dark chamber and were allowed to freely explore the apparatus for 10 min. Amounts of time spent in light and dark chambers and number of transitions were analyzed using EthoVision XT10 software (Noldus).

### Electrophysiology

Male mice at P18–26 (for mPFC measurements) and at P29–41 (for dorsolateral striatum measurements) were anesthetized with isoflurane. Mouse brain sections (300 μm) were sectioned in ice-cold dissection buffer containing (in mM) 212 sucrose, 25 NaHCO_3_, 10 D-glucose, 2 Na-pyruvate, 1.25 ascorbic acid, 1.25 NaH_2_PO_4_, 5 KCl, 3.5 MgSO_4_, and 0.5 CaCl_2_ bubbled with 95% O_2_ and 5% CO_2_ gases using Leica VT 1200 vibratome. The slices were recovered for 30 min and maintained in artificial cerebrospinal fluid (ACSF) at 32°C (in mM: 124 NaCl, 25 NaHCO_3_, 10 Glucose, 2.5 KCl, 1 NaH_2_PO_4_, 2.5 CaCl_2_, 1.3 MgSO_4_ oxygenated with 95% O_2_ and 5% CO_2_ gases). All recordings were performed after recovery for additional 30 min at room temperature. During all recordings, brain slices were maintained in a submerge-type recording chamber perfused with 27.5–28.5°C ACSF (2 ml min^–1^). Recording glass pipettes from borosilicate glass capillaries (Harvard Apparatus) were pulled using an electrode puller (Narishige). All electric responses were amplified and filtered at 2 kHz (Multiclamp 700B, Molecular Devices) and then digitized at 10 kHz (Digidata 1550, Molecular Devices). For whole-cell patch recordings in the mPFC layer 2/3 and dorsolateral striatum, a recording pipette (2.5–3.5 MΩ) was filled with the internal solution (in mM: 100 CsMeSO_4_, 10 TEA-Cl, 8 NaCl, 10 HEPES, 5 QX-314-Cl, 2 Mg-ATP, 0.3 Na-GTP and 10 EGTA with pH 7.25, 295 mOsm for mEPSCs and sEPSCs; 115 CsCl, 10 EGTA, 8 NaCl, 10 TEACl, 10 HEPES, 4 Mg-ATP, 0.3 Na-GTP, 5 QX-314 with pH 7.35, 295 mOsm for mIPSCs and sIPSC; 137 K-gluconate, 5 KCl, 10 HEPES, 0.2 EGTA, 10 Na_2_-phosphocreatine, 4 Mg-ATP, 0.5 Na-GTP with pH 7.2, 280 mOsm for excitability). To measure mEPSCs, mIPSCs, sEPSCs, and sIPSCs, mPFC layer2/3 pyramidal neurons and dorsolateral MSN neurons were voltage-clamped at −70 mV. For mEPSCs and mIPSCs, picrotoxin (60 μM) and NBQX (10 μM) + APV (50 μM) were added to ACSF with TTX (1 μM), respectively. For sEPSCs and sIPSCS, picrotoxin (60 μM) and NBQX (10 μM) + APV (50 μM) without TTX were added, respectively. mE/IPSC and sE/IPSC events were selected based on the properties of the detected currents (rise time < 1 ms, 10 pA < amplitude < 500 pA, and decay half-width > 2 ms). Responses were recorded for 2 min after maintaining stable baseline for 5 min. For neuronal excitability measurement, ACSF contained picrotoxin (60 μM), NBQX (10 μM), and AP5 (50 μM). First minimal currents were introduced to hold the membrane potential around −80 mV in a current clamp mode. To evoke depolarizing voltage sag responses, increasing amounts of depolarizing step currents (by 10 pA, −150–10 pA) were injected. Then, to elicit action potentials, increasing amounts of depolarizing currents (0–330 pA) were injected in a stepwise manner. Input resistance was calculated as the linear slope of current-voltage plots generated from a series of increasing current injection steps.

### Statistical Analysis

Statistical analyses were performed using GraphPad Prism 5 software. Details of statistical analyses are presented in Supplementary Table S2. The normality of data distributions was determined using the D’Agostino and Pearson omnibus normality test, followed by Student’s t-test (in the case of a normal distribution) and Mann–Whitney U test (in the case of a non-normal distribution). If samples were dependent on each other, a paired *t*-test (in the case of a normal distribution) or Wilcoxon signed rank test (in the case of a non-normal distribution) was used. Repeated-measures, two-way analysis of variance (ANOVA) with *post hoc* Bonferroni test (in the case of significant interactions) was used for time-varying analyses of open-field tests and Laboras tests. In cases where a Grubb’s test showed that a single significant outlier (^∗^*P* < 0.05) caused data to be non-normally distributed, the outlier value was removed prior to analysis. A one-sample *t*-test was used for the analysis of Western blot data. *P*-values < 0.05 were considered statistically significant; individual *P*-values are indicated in figures as follows: ^∗^*P* < 0.05; ^∗∗^*P* < 0.01; ^∗∗∗^*P* < 0.001; and ns, not significant.

## Results

### Generation and Basic Characterization of *Emx1-Cre;Shank3^Δ14–16^* Mice

To analyze the effects of a *Shank3* deletion restricted to glutamatergic neurons, we crossed *Shank3*^*fl/fl*^ mice (exons 14–16) with an *Emx1-Cre* mouse line, known to drive gene expression in glutamatergic neurons and glia with a dorsal telencephalic origin (Gorski et al., 2002).

**FIGURE 1:**
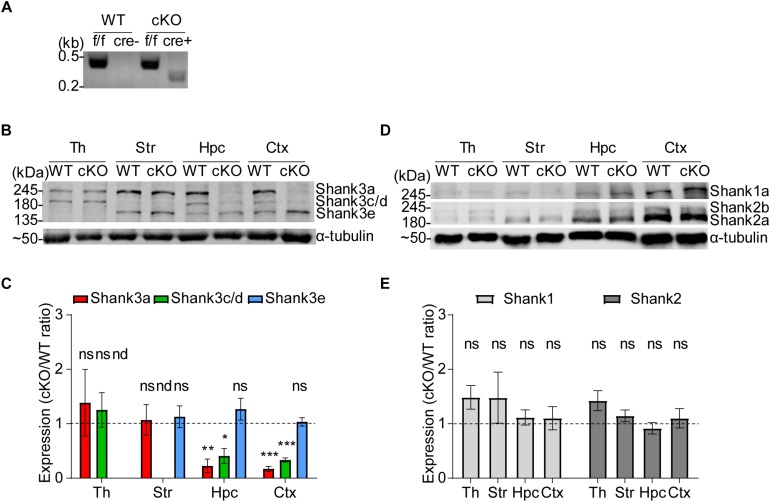
Generation and characterization of *Emx1-Cre;Shank3^Δ14–16^* mice. **(A)** PCR genotyping of *Emx1-Cre;Shank3^Δ14–16^* mice. Note that the primer set targeting exons 13 and 14 generates a PCR band for the floxed allele (478 bp), and the primer set targeting general Cre generates a PCR band (272 bp) in *Emx1-Cre;Shank3^Δ14–16^* mice, but not in WT mice. **(B,C)** Reduced levels of Shank3 protein variants in different brain regions of *Emx1-Cre;Shank3^Δ14–16^* mice (12–13 weeks, male and female). Total brain lysates were analyzed by immunoblotting using a Shank3-specific antibody (#2036) **(B)**. Neither the Shank3e isoform in the thalamus nor the Shank3c/d isoform in the striatum was quantified because of their low levels of expression in these regions. Th, thalamus; Str, striatum; Hpc, hippocampus; Ctx, cortex. cKO band signals normalized to α-tubulin are expressed relative to those from WT mice **(C)**. Data are shown as mean ± SEM. *n* = 5 pairs (WT, cKO), ^∗^*P* < 0.05, ^∗∗^*P* < 0.01, ^∗∗∗^*P* < 0.001, nd, not detectable, ns, not significant, and one sample *t*-test. **(D,E)** Normal levels of Shank1 and Shank2 protein variants in different brain regions of *Emx1-Cre;Shank3^Δ14– 16^* mice (12–13 weeks, male and female). Total brain lysates were analyzed by immunoblotting using a Shank1-specific antibody (#2100) and Shank2-specific antibody (162 202, SYSY) **(D)**. cKO band signals normalized to α-tubulin are expressed relative to those from WT mice **(E)**. Data are shown as mean ± SEM. *n* = 5 pairs (WT, cKO), ns, not significant, and one sample *t*-test.

The resulting *Emx1-Cre;Shank3^Δ14–16^* mice, genotyped by PCR (Figure 1A), exhibited strong reductions in the levels of Shank3a and Shank3c/d variants in the hippocampus and cortex (Figures 1B,C), a finding in agreement with previous results on alternative splicing in *Shank3* (Lim et al., 1999; Maunakea et al., 2010; Waga et al., 2014; Wang et al., 2014b). In contrast, *Shank3* expression was largely unaffected in the thalamus, a brain region that is minimally affected by the Emx1 driver, and the striatum, which is mainly populated by GABAergic inhibitory neurons.

Levels of other members of the Shank family of proteins, namely Shank1 (Shank1a variant reported previously) (Lim et al., 1999; Naisbitt et al., 1999) and Shank2 (Shank2a and Shank2b reported previously) (Schmeisser et al., 2012; Won et al., 2012), were unaffected by *Shank3* deletion in the tested brain regions (Figures 1D,E), indicative of the lack of compensatory changes.

### Increased Excitability in Global *Shank3^Δ14–16^* and *Emx1-Cre;Shank3^Δ14–16^* mPFC Layer 2/3 Pyramidal Neurons

Previous studies have associated *Shank3* deletion with altered neuronal excitability in human and rodent neurons (Peixoto et al., 2016; Yi et al., 2016), suggesting the possibility of altered neuronal excitability in *Shank3*-deficient cortical neurons. To determine whether *Shank3* deletion affects intrinsic excitability in layer 2/3 cortical pyramidal neurons in the mPFC, a brain region implicated in ASD, and whether glutamatergic neurons are involved, we measured and compared neuronal excitability in global *Shank3^Δ14–16^* and *Emx1-Cre;Shank3^Δ14–16^* pyramidal neurons in the prelimbic region of the mPFC.

**FIGURE 2:**
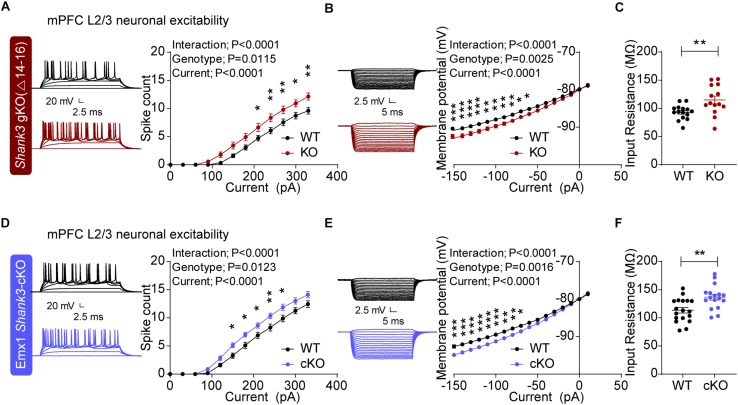
Increased excitability in global *Shank3^Δ14–16^* and *Emx1-Cre;Shank3^Δ14–16^* mPFC layer 2/3 pyramidal neurons. **(A–C)** Enhanced excitability in layer 2/3 pyramidal neurons in the prelimbic region of the mPFC in global *Shank3^Δ14–16^* mice (gKO; P20–25), as shown by current-firing curve **(A)**, current-voltage relationship **(B)** and input resistance **(C)**. Data are shown as mean ± SEM. *n* = 15 neurons from 3 mice (WT), 14, 3 (KO), ^∗^*P* < 0.05, ^∗∗^*P* < 0.01, ^∗∗∗^*P* < 0.001, repeated measures two-way ANOVA (for current-firing and current-membrane potential curves), and Student’s *t*-test (for input resistance). **(D–F)** Enhanced excitability of layer 2/3 pyramidal neurons in the prelimbic region of the mPFC in *Emx1-Cre;Shank3^Δ14–16^* mice (P20–24), as shown by current-firing curve, current-voltage relationship and input resistance. *n* = 18 neurons from 4 mice (WT), 17, 4 (cKO), ^∗^*P* < 0.05, ^∗∗^*P* < 0.01, ^∗∗∗^*P* < 0.001, repeated measures two-way ANOVA (for current-firing and current-membrane potential curves), and Student’s *t*-test (for input resistance).

Global *Shank3^Δ14–16^* mice exhibited increased neuronal excitability in layer 2/3 pyramidal neurons, as shown by the current-spike curve and input resistance, two electrophysiolgical parameters that contribute to neuronal excitability in depolarizing and hyperpolarizing ranges of membrane potentials (Figures 2A–C). *Emx1-Cre;Shank3^Δ14–16^* mice also showed similarly increased neuronal excitability in mPFC neurons (Figures 2D–F). These results suggest that glutamatergic neurons contribute to the increased neuronal excitability observed in global *Shank3^Δ14–16^* mice.

### Altered Excitatory and Inhibitory Spontaneous Synaptic Transmissions in Global *Shank3^Δ14–16^*, but Not *Emx1-Cre;Shank3^Δ14–16^*, mPFC Layer 2/3 Pyramidal Neurons

Given that neuronal excitability acts together with excitatory and inhibitory synaptic inputs to determine neuronal output function, we next measured excitatory and inhibitory synaptic transmission in *Shank3*-mutant mPFC neurons.

**FIGURE 3:**
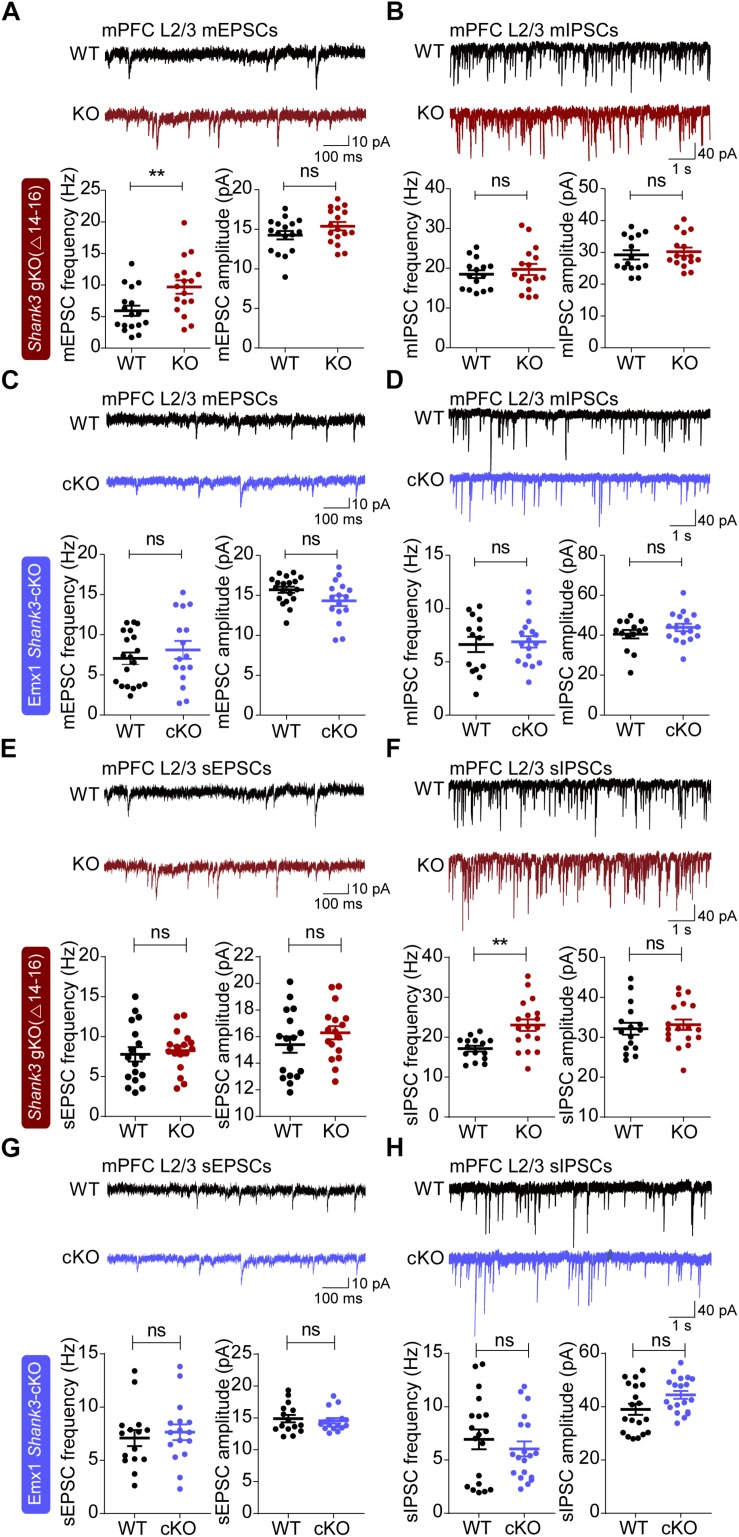
Altered excitatory and inhibitory spontaneous synaptic transmissions in global *Shank3^Δ14–16^*, but not *Emx1-Cre;Shank3^Δ14–16^*, mPFC layer 2/3 pyramidal neurons. **(A,B)** Global *Shank3^Δ14–16^* mice (P21–26) show increased frequency, but normal amplitude, of mEPSCs and normal mIPSCs in layer 2/3 pyramidal neurons in the prelimbic region of the mPFC. Data are shown as mean ± SEM. *n* = 17 neurons from 5 mice (WT), 17, 7 (KO) for mEPSCs, 15, 5 (WT), 16, 6 (KO) for mIPSCs, ^∗∗^*P* < 0.01, ns, not significant, Student’s *t*-test. **(C,D)** *Emx1-Cre;Shank3^Δ14–16^* mice (P18–22) show normal mEPSCs and mIPSCs in layer 2/3 pyramidal neurons in the prelimbic region of the mPFC. *n* = 19, 4 (WT), 16, 4 (cKO) for mEPSCs, 14, 4 (WT), 17, 4 (cKO) for mIPSCs, Student’s *t*-test (amplitude of mEPSC and frequency of mIPSC), and Mann–Whitney U test (frequency of mEPSC and amplitude of mIPSC). **(E,F)** Global *Shank3^Δ14–16^* mice (P21–24) show normal sEPSCs and increased frequency, but normal amplitude, of sIPSCs in layer 2/3 pyramidal neurons in the prelimbic region of the mPFC. Data are shown as mean ± SEM. *n* = 17 neurons from 3 mice (WT), 17, 3 (KO) for sEPSCs, 15, 3 (WT), 18, 3 (KO) for sIPSCs, ^∗∗^*P* < 0.01, ns, not significant, Student’s *t*-test. **(G,H)** *Emx1-Cre;Shank3^Δ14–16^* mice (P20–25) show normal sEPSCs and sIPSCs in layer 2/3 pyramidal neurons in the prelimbic region of the mPFC. *n* = 15, 3 (WT), 16, 3 (cKO) for sEPSCs,19, 3 (WT), 19, 3 (cKO) for sIPSCs, ns, not significant, Student’s *t*-test (frequency and amplitude of sEPSC and frequency of sIPSC), and Mann–Whitney U test (amplitude of sIPSC).

The frequency, but not amplitude, of miniature excitatory postsynaptic currents (mEPSCs) was increased in the prelimbic region of the mPFC of global *Shank3^Δ14–16^* layer 2/3 neurons compared with WT mice (Figure 3A). In contrast to mEPSCs, miniature inhibitory postsynaptic currents (mIPSCs) were not changed in global *Shank3^Δ14–16^* mice (Figure 3B).

In *Emx1-Cre;Shank3^Δ14–16^* layer 2/3 mPFC neurons, both mEPSCs and mIPSCs were normal (Figures 3C,D). This suggests that excitatory neurons are less likely to contribute to the increased mEPSC frequency observed in global *Shank3^Δ14–16^* mPFC neurons.

We also measured excitatory and inhibitory synaptic transmission in the presence of network activity by excluding tetrodotoxin (a blocker of action potential firing) during slice recordings. The frequency and amplitude of spontaneous EPSCs (sEPSCs) were normal in global *Shank3^Δ14–16^* layer 2/3 pyramidal neurons in the prelimbic region of the mPFC compared with those in WT neurons (Figure 3E).

Notably, the frequency, but not amplitude, of spontaneous IPSCs (sIPSCs) was increased in global *Shank3^Δ14–16^* layer 2/3 pyramidal neurons (Figure 3F). In addition, both sEPSCs and sIPSCs were normal in *Emx1-Cre;Shank3^Δ14–16^* mice (Figures 3G,H). These results collectively suggest that global *Shank3* deletion leads to increases in mEPSC frequency and sIPSC frequency, whereas glutamatergic *Shank3* deletion has no effects on any forms of spontaneous synaptic transmission in layer 2/3 mPFC pyramidal neurons.

### Normal Spontaneous Excitatory and Inhibitory Synaptic Transmission in *Emx1-Cre;Shank3^Δ14–16^* Dorsolateral Striatal Neurons

Dysfunctions in striatal regions, where Shank3 is strongly expressed (Peca et al., 2011), have been associated with abnormal behaviors in *Shank3*-mutant mice (Monteiro and Feng, 2017). Because our previous results revealed decreased excitatory synaptic transmission in dorsolateral striatal neurons in both global *Shank3^Δ14–16^* and *Viaat-Cre;Shank3^Δ14–16^* mice (Yoo et al., 2018), we next measured spontaneous excitatory and inhibitory synaptic transmission in dorsolateral striatal neurons.

**FIGURE 4:**
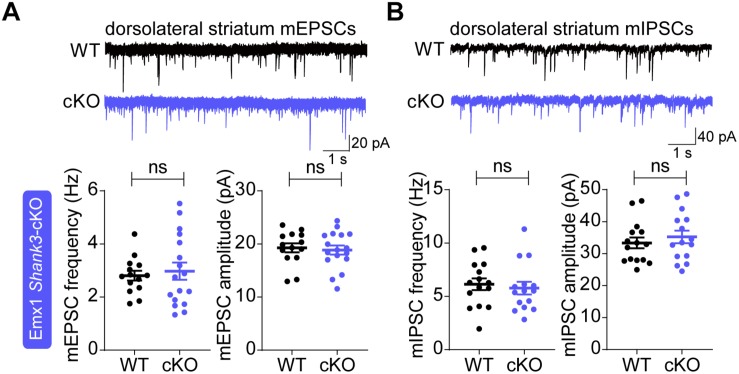
Normal spontaneous excitatory and inhibitory synaptic transmission in *Emx1-Cre;Shank3^Δ14–16^* dorsolateral striatal neurons. **(A)** *Emx1-Cre;Shank3^Δ14–16^* mice (P29–35) show normal frequency and amplitude of mEPSCs in dorsolateral striatal neurons. Data are shown as mean ± SEM. *n* = 14 neurons from 3 mice (WT), 17, 3 (cKO), ns, not significant, Student’s *t*-test. **(B)** *Emx1-Cre;Shank3^Δ14–16^* mice (P34–41) show normal frequency and amplitude of mIPSCs in dorsolateral striatal neurons. *n* = 15, 3 (WT), 15, 3 (cKO), ns, not significant, Student’s *t*-test.

However, there were no changes in the frequency or amplitude of mEPSCs in *Emx1-Cre;Shank3^Δ14–16^* dorsolateral striatal neurons compared with WT neurons (Figure 4A). In addition, neither the frequency nor amplitude of mIPSCs was altered in *Emx1-Cre;Shank3^Δ14–16^* dorsolateral striatal neurons (Figure 4B). These results contrast with the strongly decreased mEPSC frequency and amplitude in dorsolateral striatal neurons in global *Shank3^Δ14–16^* and *Viaat-Cre;Shank3^Δ14–16^* mice (Yoo et al., 2018).

### Enhanced Direct Social Interaction, but Normal Social Approach and Social Communication, in *Emx1-Cre;Shank3^Δ14–16^* Mice

Given the well-known association between *SHANK3* and various neurodevelopmental disorders, including ASD, PMS and schizophrenia (Phelan and McDermid, 2012; Monteiro and Feng, 2017), we first subjected *Emx1-Cre; Shank3^Δ14–16^* mice to behavioral tests in the social domain.

**FIGURE 5:**
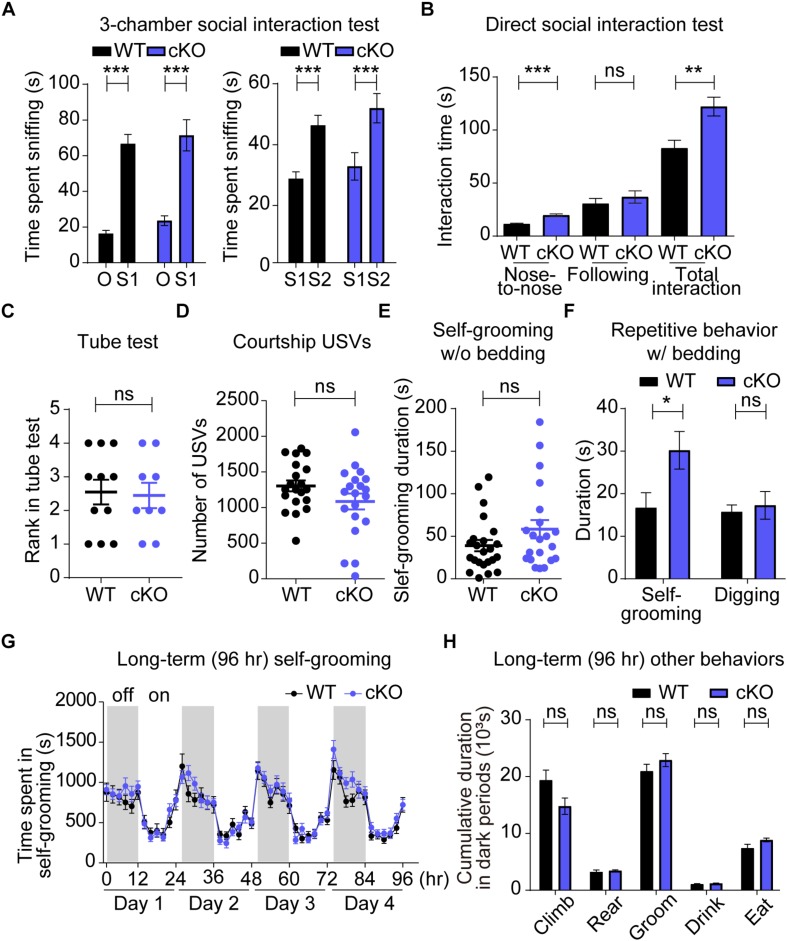
Enhanced direct social interaction and repetitive self-grooming in *Emx1-Cre;Shank3^Δ14–16^* mice. **(A–D)** *Emx1-Cre;Shank3^Δ14–16^* mice (**A**, 13–20 weeks; **B**, 15–21 weeks; **C**, 16–32 weeks; **D**, 15–21 weeks) show normal social approach in the three-chamber test **(A)**, enhanced direct social interaction **(B)**, normal social dominance in the tube test **(C)**, and normal courtship USVs **(D**; *P* = 0.088). Data are shown as mean ± SEM. *n* = 23 (WT), 20 (cKO) for three-chamber, 10 (WT), 9 (cKO) for direct social interaction, 11 (WT), 9 (cKO) for tube test, and 20 (WT), 20 (cKO) for USV, ^∗∗^*P* < 0.01, ^∗∗∗^*P* < 0.001, paired *t*-test (WT S1-O, WT S1-S2, and cKO S1-S2), Wilcoxon signed rank test (cKO S1-O), and Student’s *t*-test (for the direct social interaction test, and adult USV test). **(E–H)** *Emx1-Cre;Shank3^Δ14–16^* mice (**E**, 12–17 weeks; **F**, 12–18 weeks; **G,H**, 10–17 weeks) show enhanced self-grooming and normal digging in home cages with bedding **(F)**, but normal self-grooming in a novel home cage without bedding **(E)** and in Laboras cages **(G,H)**. Data are shown as means ± SEM; data values in panel H represent those from light-off periods (shaded durations). *n* = 24 (WT), 21 (cKO) for w/o bedding, 16 (WT), 14 (cKO) for w/bedding, and 13 (WT), 14 (cKO) for Laboras, ^∗^*P* < 0.05, repeated measures two-way ANOVA (for Laboras, left panel; genotype *p*-value = 0.3944), Student’s *t*-test [for digging time of repetitive behavior and Laboras (climbing, rearing, grooming, and eating)], and Mann–Whitney U test [for self-grooming test, self-grooming time of repetitive behavior, and Laboras (drinking)].

In the three-chambered social interaction test, designed to measure social approach and social novelty recognition (Crawley, 2004; Moy et al., 2004; Silverman et al., 2010), *Emx1-Cre;Shank3^Δ14–16^* mice showed social approach behaviors that are comparable to those of WT mice, as shown by time spent sniffing social and object targets (Figure 5A). In addition, *Emx1-Cre;Shank3^Δ14–16^* mice displayed normal social novelty recognition, as shown by time spent sniffing familiar and novel stranger mice.

Intriguingly, in experiments using genotype- and age-matched mouse pairs, *Emx1-Cre;Shank3^Δ14–16^* mice showed enhanced social interaction in the direct social interaction test, as shown by time spent in nose-to-nose sniffing and total social interaction (Figure 5B). These results indicate that *Emx1-Cre;Shank3^Δ14–16^* mice display normal social approach and social novelty recognition, but abnormally enhanced direct social interaction, similar to the social behaviors of global *Shank3^Δ14–16^* and *Viaat-Cre;Shank3^Δ14–16^* mice in these tests (Yoo et al., 2018). These changes do not seem to involve altered social dominance, as supported by the lack of genotype difference in the Tube test (Figure 5C). These results suggest that both glutamatergic and GABAergic neurons contribute to the abnormally enhanced direct social interaction in global *Shank3^Δ14–16^* mice.

We next evaluated USVs, which are strongly associated with rodent behaviors and emotional states, including social communication (Knutson et al., 1998, 2002; Portfors, 2007; Scattoni et al., 2009). Adult male *Emx1-Cre;Shank3^Δ14–16^* mice encountering a novel female mouse emitted normal numbers of USVs compared with WT mice (Figure 5D). Notably, this result differs from the suppressed courtship USVs observed in global *Shank3^Δ14–16^* and *Viaat-Cre;Shank3^Δ14–16^* mice (Yoo et al., 2018), suggesting that GABAergic, but not glutamatergic, neurons strongly contribute to the USV deficits in global *Shank3^Δ14–16^* mice.

### Modestly Enhanced Repetitive Self-Grooming, but Normal Digging, in *Emx1-Cre;Shank3^Δ14–16^* Mice

We next evaluated repetitive behaviors, a core component of ASD-related behavior, in *Emx1-Cre;Shank3^Δ14–16^* mice. *Emx1-Cre;Shank3^Δ14–16^* mice displayed enhanced self-grooming in a new home cage with bedding but normal self-grooming in a new home cage without bedding (Figures 5E,F), suggesting that the presence of bedding is required for repetitive behavior in addition to a new cage or environment. This result shows similarities to the strong self-grooming behaviors in global *Shank3^Δ14–16^* mice observed in all three environments (new home cage with bedding, new home cage without bedding, and Laboras cages), but is more comparable to the mildly enhanced self-grooming in *Viaat-Cre;Shank3^Δ14–16^* mice, observed only in a new home cage with bedding (Yoo et al., 2018). Measurements of digging, another method for quantifying repetitive behavior, showed no changes in *Emx1-Cre;Shank3^Δ14–16^* mice compared with WT mice, even in the presence of bedding. This contrasts with the decreased digging observed in both global *Shank3^Δ14–16^* and *Viaat-Cre;Shank3^Δ14–16^* mice (Yoo et al., 2018).

*Emx1-Cre;Shank3^Δ14–16^* mice subjected to the Laboras test, designed to measure mouse behaviors for a long period of time (i.e., four consecutive days) in a light/dark-cycling environment with bedding (Quinn et al., 2003, 2006), showed normal levels of self-grooming (Figures 5G,H). The results of these tests, in which mice were fully habituated, especially on days 2–4, suggest that *Emx1-Cre;Shank3^Δ14–16^* mice show enhanced self-grooming only in a particular environment (i.e., novel home cage with bedding). Other behaviors of *Emx1-Cre;Shank3^Δ14–16^* mice, including climbing, rearing, drinking and eating, were unchanged in Laboras cages (Figure 5H).

### Normal Locomotor Activity and Enhanced Anxiolytic-Like Behavior in *Emx1-Cre;Shank3^Δ14–16^* Mice

**FIGURE 6:**
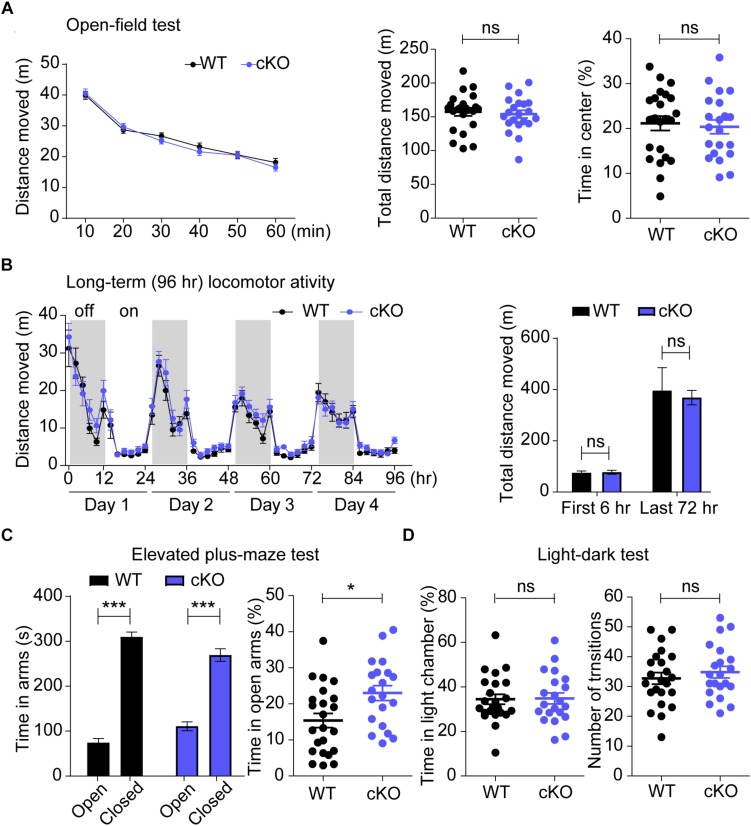
Normal locomotor activity in open-field and Laboras cages, and enhanced anxiety-like behavior in elevated plus-maze (EPM) in *Emx1-Cre;Shank3^Δ14–16^* mice. **(A,B)** *Emx1-Cre;Shank3^Δ14–16^* mice **(A**, 11–17 weeks; **B**, 10–17 weeks) show normal levels of locomotor activity in the open-field test **(A)** and in Laboras cages **(B)**. Note that these mice spend a normal amount of time in the center region of the open-field arena, indicative of normal anxiety-like behavior in this test. Data are shown as means ± SEM; data values in the bar graph in panel B represent those from light-off periods (shaded durations). *n* = 23 (WT), 21 (cKO) for open-field and 13 (WT), 14 (cKO) for Laboras, repeated measures of two-way ANOVA (for the left panels in open-field and Laboras; genotype *p*-values = 0.6889 and 0.3025, respectively), Student’s *t*-test (for the right panels in open-field and first 6 h of the right panels in Laboras), and Mann–Whitney U test (for the last 72 h of the right panels in Laboras). **(C)** *Emx1-Cre;Shank3^Δ14–16^* mice (14–19 weeks) spend an increased amount of time in the open arm of the elevated plus-maze (EPM). *n* = 23 (WT), 20 (cKO), ^∗^*P* < 0.05, ^∗∗∗^*P* < 0.001, Student’s *t*-test (right panels of elevated plus maze test) and paired *t*-test (for left panels of elevated plus maze test). **(D)** *Emx1-Cre;Shank3^Δ14–16^* mice (14–19 weeks) spend a normal amount of time in the light chamber of the light-dark (LD) apparatus. *n* = 23 (WT), 21 (cKO), ns, not significant, Student’s *t*-test.

Because hyperactivity and anxiety are observed in ASD, PMS and schizophrenia, we also evaluated locomotor activities of *Emx1-Cre;Shank3^Δ14–16^* mice. In the open-field test, representing a novel environment, *Emx1-Cre;Shank3^Δ14–16^* mice showed normal levels of locomotor activity, as shown by distance moved during 60 min (Figure 6A). In Laboras cages, representing a familiar environment, *Emx1-Cre;Shank3^Δ14–16^* mice showed normal levels of locomotor activities during the last 72 h (Figure 6B). Locomotion in Laboras cages was also unchanged during the first 6 h, similar to the results of the open-field test. These results suggest that glutamatergic *Shank3* deletion does not affect locomotor activity, in contrast to the reported hypoactivity of both global *Shank3^Δ14–16^* and *Viaat-Cre;Shank3^Δ14–16^* mice (Yoo et al., 2018).

In anxiety-related behavioral tests, *Emx1-Cre;Shank3^Δ14–16^* mice spent a normal amount of time in the center region of the open-field arena (Figure 6A), but spent an increased amount of time in the open arm of the elevated plus-maze (EPM) (Figure 6C), and a normal amount of time in the light chamber of the LD apparatus (Figure 6D). The normal open-field center time and increased EPM open-arm time in *Emx1-Cre;Shank3^Δ14–16^* mice are similar to behaviors observed in global *Shank3^Δ14–16^* mice, but differ from the decreased open-field center time and normal EPM open-arm time observed in *Viaat-Cre;Shank3^Δ14–16^* mice (Yoo et al., 2018). In addition, the normal light-chamber time in the LD test in *Emx1-Cre;Shank3^Δ14–16^* mice differs from the reduced light-chamber time (anxiety-like behavior) in global *Shank3^Δ14–16^* and *Viaat-Cre;Shank3^Δ14–16^* mice (Yoo et al., 2018) (summarized in Table 1). Therefore, the two contrasting anxiety-like behaviors in global *Shank3^Δ14–16^* mice—anxiolytic-like behavior in the EPM and anxiety-like behavior in the LD apparatus—seem to more strongly involve glutamatergic and GABAergic neurons, respectively.

**TABLE 1:** Comparison of electrophysiological and behavioral phenotypes of global *Shank3^Δ14–16^*, *Emx1-Cre;Shank3^Δ14–16^*, and *Viaat-Cre;Shank3^Δ14–16^* mice.

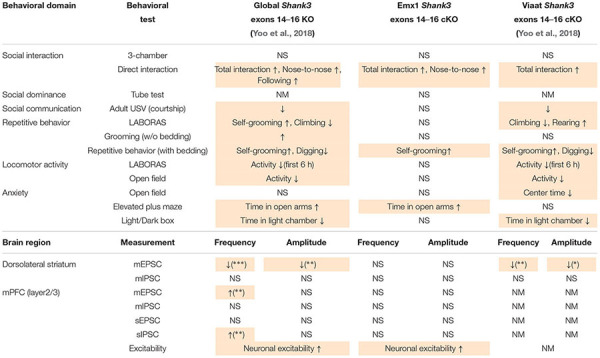

*Table summarizes increases or decreases in various electrophysiological and behavioral phenotypes in a given mouse line relative to WT/control mice, but is not intended to compare phenotypic severities across different mouse lines. ^∗^*P* < 0.05, ^∗∗^*P* < 0.01, and ^∗∗∗^*P* < 0.001; NS, no significant change; NM, not measured; up and down arrows, increases and decreases, respectively. Areas shaded in orange represent similar phenotypes.*

### Control *Emx1-Cre* Mice Show Normal Locomotor Activity, Anxiety-Like Behavior, Direct Social Interaction, and Repetitive Behavior

It is conceivable that control mice harboring *Emx1-Cre* alone might show behavioral abnormalities. To test this, we analyzed the behaviors of *Emx1-Cre* mice. These mice showed normal behaviors in Laboras cages, including locomotion, climbing, and rearing (Supplementary Figure S1A). In addition, *Emx1-Cre* mice showed normal levels of locomotor activity in the open-field test and time spent in the center region of the open-field arena (Supplementary Figure S1B). These mice also showed normal levels of time spent in the open arm of the EPM (Supplementary Figure S1C), direct social interaction (Supplementary Figure S1D), and self-grooming and digging in home cages with bedding (Supplementary Figure S1E). These results suggest that control *Emx1-Cre* mice show normal locomotion, repetitive behavior, and anxiety-related behaviors.

## Discussion

In this study, we investigated the impacts of glutamatergic *Shank3* (exons 14–16) deletion and compared them with those observed in global *Shank3^Δ14–16^* and *Viaat-Cre;Shank3^Δ14–16^* mice. Our data indicate that the synaptic/neuronal phenotypes of *Emx1-Cre;Shank3^Δ14–16^* mice were similar in part to those observed in global *Shank3^Δ14–16^* mice (summarized in Table 1). Moreover, social and repetitive behavioral deficits were similar between *Emx1-Cre;Shank3^Δ14–16^* and *Viaat-Cre;Shank3^Δ14–16^* mice, suggesting that these behaviors involve shared contributions of glutamatergic and GABAergic neurons. However, electrophysiological and behavioral phenotypes of *Emx1-Cre;Shank3^Δ14–16^* and *Viaat-Cre;Shank3^Δ14–16^* mice were largely distinct (Table 1).

Electrophysiologically, *Emx1-Cre;Shank3^Δ14–16^* layer 2/3 pyramidal neurons in the prelimbic region of the mPFC showed increased neuronal excitability (Figures 2D–F). The fact that neuronal excitability was similarly increased in global *Shank3^Δ14–16^* layer 2/3 neurons (Figures 2A–C) suggests that glutamatergic neurons strongly contribute to the increased excitability in global *Shank3^Δ14–16^* layer 2/3 neurons. Importantly, a previous study reported that neuronal excitability is increased in human neurons harboring a *SHANK3* (exon 13) deletion, in association with increased input resistance and decreased hyperpolarization-activated cation currents (*I*_h_). This latter effect is mediated by hyperpolarization-activated cyclic nucleotide-gated (HCN) channels, which directly interact with Shank3 (Yi et al., 2016). In addition, cultured hippocampal neurons from *Shank3*-deficient mice (exons 13–16) (Peca et al., 2011) display similar increases in neuronal excitability (Yi et al., 2016). These results, together with our demonstration of increased neuronal excitability in global *Shank3^Δ14–16^* and *Emx1-Cre;Shank3^Δ14–16^* mPFC layer 2/3 neurons, collectively suggest that increased neuronal excitability represents a conserved mechanism underlying *Shank3* deletion-induced behavioral abnormalities.

In terms of synaptic transmission, neither mEPSCs or mIPSCs in the mPFC were changed in *Emx1-Cre;Shank3^Δ14–16^* layer 2/3 pyramidal neurons (Figures 3C,D). This contrasts with the increased frequency, but not amplitude, of mEPSCs in global *Shank3^Δ14–16^* layer 2/3 pyramidal neurons (Figures 3A,B). Therefore, the increased mEPSC frequency in global *Shank3^Δ14–16^* layer 2/3 pyramidal neurons may not involve changes in glutamatergic neurons, thus implying non-cell-autonomous mechanisms. Indeed, this possibility of non-cell-autonomous mechanisms is in agreement with the known roles of Shank3 as a key component of excitatory postsynaptic compartments (Sheng and Kim, 2000, 2011; Sheng and Sala, 2001; Boeckers et al., 2002; Sheng and Hoogenraad, 2007; Grabrucker et al., 2011; Jiang and Ehlers, 2013; Sala et al., 2015; Monteiro and Feng, 2017; Mossa et al., 2017).

Then how might a *Shank3* deletion lead to an increase in mEPSC frequency in global *Shank3^Δ14–16^* layer 2/3 pyramidal neurons? Increased mEPSC frequency may involve increased excitatory synapse number or increased excitatory synaptic transmission through mechanisms, including increased neuronal excitability of presynaptic neurons and increased efficiency of presynaptic release. Therefore, one possibility is that the increased neuronal excitability in global *Shank3^Δ14–16^* mPFC neurons increases the output function of these neurons and activates the intra-cortical network between layer 2/3 neurons, promoting the development of excitatory synapses in target layer 2/3 cortical neurons. Intriguingly, a previous study has shown that Shank3 could be detected in axonal compartments and nerve terminals and negative regulates presynaptic NMDARs (Halbedl et al., 2016), suggesting that the loss of presynaptic Shank3 might contribute to the increased mEPSC frequency. Alternatively, the increased neuronal excitability induced by loss of the interaction between Shank3 and HCN channels (Yi et al., 2016) may promote excitatory synaptic transmission and excitatory synapse development in a cell-autonomous manner; however, this is an unlikely possibility, as noted above.

Our measurements of sEPSCs and sIPSCs provide additional insight into the role of network activity in the context of a *Shank3* deletion. Specifically, global *Shank3^Δ14–16^* layer 2/3 pyramidal neurons showed normalized sEPSC frequency and increased sIPSC frequency (Figures 3E,F), findings that contrast with the increased mEPSC frequency and normal mIPSC frequency in the same neurons (Figures 3A,B). These sEPSC/sIPSC phenotypes likely represent compensatory changes that serve to suppress the increased mEPSC frequency as well as the increased neuronal excitability in these neurons and thus normalize the neuronal output. Indeed, the fact that sEPSCs in global *Shank3^Δ14–16^* layer 2/3 pyramidal neurons are normalized suggests that these compensatory changes could actually normalize the neuronal output, at least in the slice preparation, which likely represents baseline conditions. However, the consequence of these compensatory effects seems to be abnormally increased inhibitory synaptic transmission onto pyramidal neurons that still maintain their increased neuronal excitability, as measured in the presence of network activity. Therefore, although neuronal output was apparently normalized in layer 2/3 neurons, the balance between excitatory and inhibitory synaptic transmission, and neuronal activity, might be disrupted. In keeping with this, an imbalance in excitation/inhibition ratio has been implicated in ASD (Rubenstein and Merzenich, 2003; Yizhar et al., 2011; Lee et al., 2015; Nelson and Valakh, 2015; Lee E. et al., 2017; Selimbeyoglu et al., 2017). A disruption in excitation/inhibition balance may also alter network properties such as brain rhythms. Indeed, altered EEG rhythms, including an increase in gamma power, have been observed in *Shank3*-mutant mice (Han et al., 2013; Wang et al., 2016; Dhamne et al., 2017; Ingiosi et al., 2019; Yoo et al., 2019) as well as in *SHANK3*-related ASD and PMS (Soorya et al., 2013; Holder and Quach, 2016).

Behaviorally, glutamatergic and GABAergic *Shank3* deletions seem to differentially contribute to the abnormal behaviors observed in global *Shank3^Δ14–16^* mice. For instance, increased direct social interaction and normal social approach were observed in all three mouse lines (global, Emx1, and Viaat) (Figures 5A,B). Enhanced self-grooming was also observed in the three mouse lines (Figures 5E–H), although it was stronger in global *Shank3^Δ14–16^* mice. These results suggest that both glutamatergic and GABAergic neurons contribute to the social and repetitive behavioral abnormalities in *Emx1-Cre;Shank3^Δ14–16^* mice. One behavior that deviated from this shared contribution of glutamatergic and GABAergic neurons was courtship USVs, which were normal in *Emx1-Cre;Shank3^Δ14–16^* mice, but suppressed in global *Shank3^Δ14–16^* and *Viaat-Cre;Shank3^Δ14–16^* mice (Figure 5D). In addition, anxiolytic-like (EPM open-arm time) and anxiety-like (LD light-chamber time) behaviors in global *Shank3^Δ14–16^* mice were observed selectively in *Emx1-Cre;Shank3^Δ14–16^* and *Viaat-Cre;Shank3^Δ14–16^* mice, respectively (Figures 6C,D).

Identifying the brain regions and circuit mechanisms underlying this differential recapitulation of behavioral phenotypes of global *Shank3^Δ14–16^* mice in *Emx1-Cre;Shank3^Δ14–16^* and *Viaat-Cre;Shank3^Δ14–16^* mice could be a highly speculative undertaking. However, one brain region that is strongly associated with Shank3-dependent social and repetitive behavioral deficits is the striatum (Peca et al., 2011; Filice et al., 2016; Fuccillo, 2016; Jaramillo et al., 2016, 2017; Mei et al., 2016; Peixoto et al., 2016; Wang et al., 2016, 2017; Zhou et al., 2016; Lee Y. et al., 2017; Reim et al., 2017; Vicidomini et al., 2017; Bey et al., 2018; Fourie et al., 2018; Yoo et al., 2018). More recently, the ventral striatum and its upstream regions (ventral tegmental area and dorsal raphe nucleus) have been implicated in the social deficits in *Shank3*-mutant mice (Bariselli et al., 2016, 2018; Bariselli and Bellone, 2017; Luo et al., 2017). Intriguingly, however, our electrophysiological results indicated that *Emx1-Cre;Shank3^Δ14–16^* mice do not display altered excitatory or inhibitory synaptic transmission in dorsolateral striatal neurons (Figure 4), a finding that strongly contrasts with the reduced excitatory synaptic transmission in dorsolateral striatal neurons in global *Shank3^Δ14–16^* and *Viaat-Cre;Shank3^Δ14–16^* mice (Yoo et al., 2018). Therefore, potential alterations in excitatory synapses in prefrontal cortical neurons in *Emx1-Cre;Shank3^Δ14–16^* mice do not seem to affect excitatory afferents to dorsal striatal neurons. We thus hypothesize that glutamatergic and GABAergic neurons contribute to the social or repetitive behavioral deficits through distinct synapses, circuits and mechanisms, at least in the dorsal striatum.

Notably, a recent study on *Shank3^Δ4–22^* mice carrying deletions in exons 4–22 (not exons 14–16, as in the current study) reported behavioral phenotypes that are surprisingly similar to those observed in our global *Shank3^Δ14–16^* mice (Yoo et al., 2018), including normal social approach, enhanced direct social interaction, suppressed courtship USVs, enhanced self-grooming, open-field hypoactivity, and anxiolytic-like behavior (EPM) (Wang et al., 2016). In addition, a more recent related study investigated the impacts of a *Shank3* (exons 4–22) deletion restricted to Nex-positive glutamatergic neurons in the cortex, hippocampus, and amygdala (*Nex-Cre;Shank3^Δ4–22^* mice) (Bey et al., 2018). Intriguingly, *Nex-Cre;Shank3^Δ4–22^* mice recapitulated many behavioral phenotypes of global *Shank3^Δ4–22^* mice, including normal social approach and enhanced self-grooming, similar to the results from global *Shank3^Δ14–16^* and *Emx1-Cre;Shank3^Δ14–16^* mice reported here. In addition, these *Nex-Cre;Shank3^Δ4–22^* mice did not recapitulate the suppressed courtship USV or hypoactivity phenotypes of global *Shank3^Δ4–22^* mice. Again, this is similar to the results from our mice (global and Emx1), which together with our demonstration that *Viaat-Cre;Shank3^Δ14–16^* mice display suppressed courtship USVs suggests (Yoo et al., 2018) that GABAergic neurons may be important for the courtship USV phenotype in *Shank3*-deficient mice.

However, *Nex-Cre;Shank3^Δ4–22^* mice not only failed to recapitulate the hypoactivity of global *Shank3^Δ4–22^* mice, they actually showed increased locomotor activity in open-field tests (Bey et al., 2018), results in contrast with the normal locomotor activity behavior in our *Emx1-Cre;Shank3^Δ14–16^* mice. Whether these differences involve differentially altered striatal synaptic transmission remains unclear because the previous study on *Nex-Cre;Shank3^Δ4–22^* mice analyzed synaptic transmission only in the hippocampus (Bey et al., 2018). However, these discrepancies could be attributable to differences in the specific exons of *Shank3* deleted or specific characteristics of *Nex-Cre* versus *Emx1-Cre* mice (Guo et al., 2000; Gorski et al., 2002; Goebbels et al., 2006).

In conclusion, our results suggest that glutamatergic *Shank3* (exons 14–16) deletion increases neuronal excitability in layer 2/3 mPFC cortical neurons, but has no effect on synaptic transmission in dorsal striatal neurons. It also induces social and repetitive behavioral deficits, similar to the effects of global and GABAergic *Shank3* deletions.

## Data Availability Statement

The raw data supporting the conclusions of this manuscript will be made available by the authors, without undue reservation, to any qualified researcher.

## Ethics Statement

The animal study was reviewed and approved by the Committee of Animal Research at Korea Advanced Institute of Science and Technology (KAIST) (KA2016-30).

## Author Contributions

TY, HC, and JL performed the behavioral experiments. TY performed the immunoblot experiments. TY, HP, and HC performed the electrophysiological experiments. TY and EK designed the experiments and wrote the manuscript.

## Conflict of Interest

The authors declare that the research was conducted in the absence of any commercial or financial relationships that could be construed as a potential conflict of interest.

## Supplementary Material

FIGURE S1 Normal locomotor activity, anxiety-like behavior, direct social interaction, and repetitive behavior in control *Emx1-Cre* mice. **(A)** *Emx1-Cre* mice (11–12 weeks) show normal behaviors in Laboras cages. Data are shown as mean ± SEM. *n* = 19 (WT), 15 (Emx1-Cre), repeated measures two-way ANOVA (for the left panel; genotype *p*-values = 0.3600), Student’s *t*-test (distance moved of last 72 h, climbing, rearing, drinking, and eating), Mann–Whitney U test (distance moved of first 6 h and grooming). **(B)** *Emx1-Cre* mice (10–12 weeks) show normal locomotor activity and time spent in the center region in the open-field test. *n* = 19 mice (WT), 16 (Emx1-Cre), repeated measures two-way ANOVA (for the left panel; genotype *p*-values = 0.9795), Student’s *t*-test. **(C)** *Emx1-Cre* mice (11–12 weeks) show normal anxiety-like behavior in the elevated plus-maze (EPM). *n* = 18 (WT), 16 (Emx1-Cre), ^∗∗∗^*P* < 0.001, paired *t*-test (for the left panels), and Student’s *t*-test (for the right panel). **(D)** *Emx1-Cre* mice (21–30 weeks) show normal levels of direct social interaction compared with sex- and age-matched conspecific mice. *n* = 7 (WT), 5 (Emx1-Cre), Mann–Whitney U test. **(E)** Emx1-Cre mice (11–16 weeks) show normal self-grooming and digging in home cages with bedding. *n* = 19 (WT), 16 (Emx1-Cre), Student’s *t*-test (duration of self-grooming), and Mann–Whitney U test (duration of digging).

TABLE S1 The order of behavior experiments.

TABLE S2 Statistics table.
