# Supplementary figures and images for: Shank3 Exons 14–16 Deletion in Glutamatergic Neurons Leads to Social and Repetitive Behavioral Deficits Associated With Increased Cortical Layer 2/3 Neuronal Excitability
Source: Front Cell Neurosci. 2019 Oct 10;13:458. doi: 10.3389/fncel.2019.00458 (PMC6795689; doi:10.3389/fncel.2019.00458)

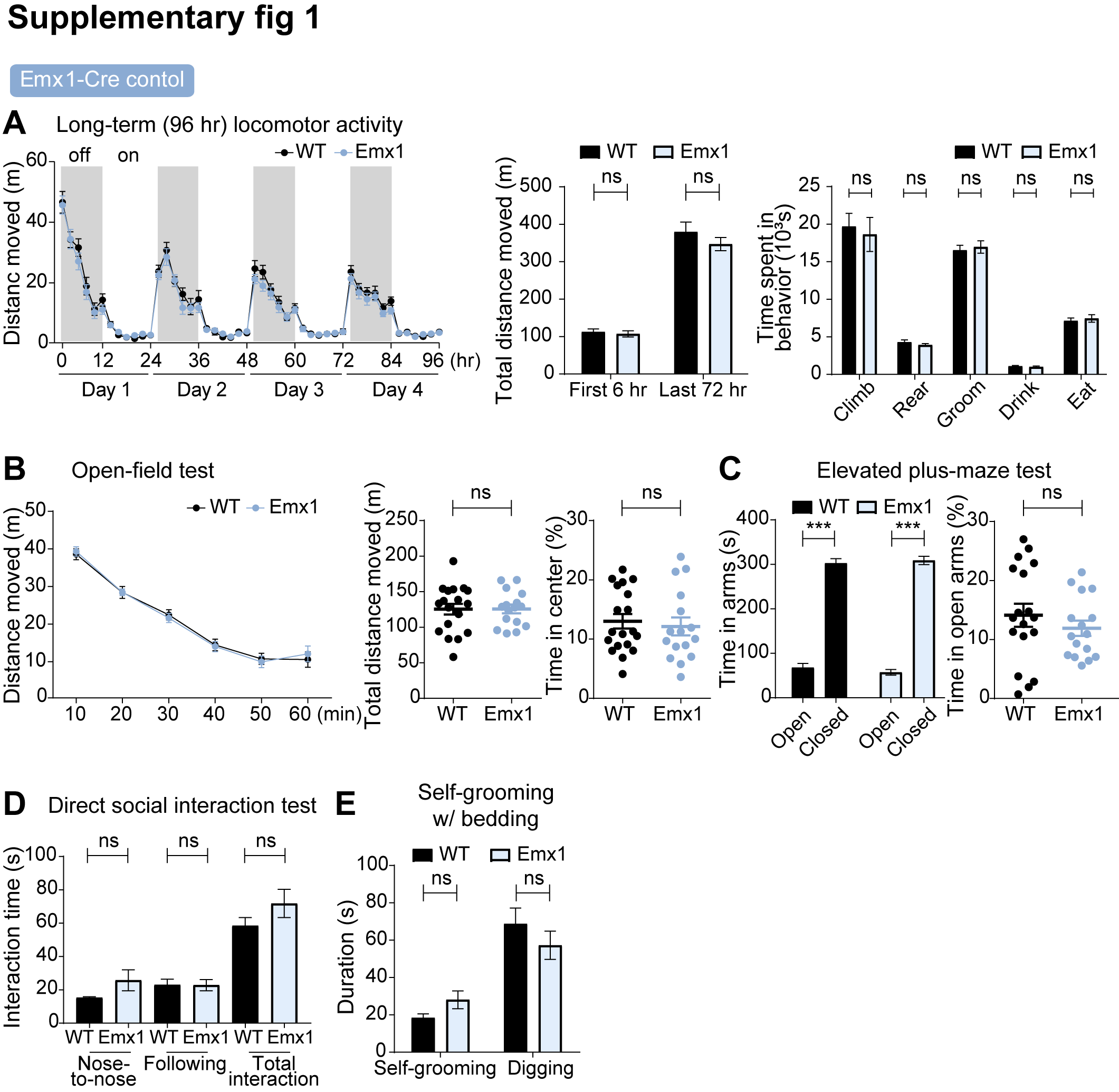

Supplement: FIGURE S1 — Normal locomotor activity, anxiety-like behavior, direct social interaction, and repetitive behavior in control Emx1-Cre mice. (A) Emx1-Cre mice (11–12 weeks) show normal behaviors in Laboras cages. Data are shown as mean ± SEM. n = 19 (WT), 15 (Emx1-Cre), repeated measures two-way ANOVA (for the left panel; genotype p-values = 0.3600), Student’s t-test (distance moved of last 72 h, climbing, rearing, drinking, and eating), Mann–Whitney U test (distance moved of first 6 h and grooming). (B) Emx1-Cre mice (10–12 weeks) show normal locomotor activity and time spent in the center region in the open-field test. n = 19 mice (WT), 16 (Emx1-Cre), repeated measures two-way ANOVA (for the left panel; genotype p-values = 0.9795), Student’s t-test. (C) Emx1-Cre mice (11–12 weeks) show normal anxiety-like behavior in the elevated plus-maze (EPM). n = 18 (WT), 16 (Emx1-Cre), ∗∗∗P < 0.001, paired t-test (for the left panels), and Student’s t-test (for the right panel). (D) Emx1-Cre mice (21–30 weeks) show normal levels of direct social interaction compared with sex- and age-matched conspecific mice. n = 7 (WT), 5 (Emx1-Cre), Mann–Whitney U test. (E) Emx1-Cre mice (11–16 weeks) show normal self-grooming and digging in home cages with bedding. n = 19 (WT), 16 (Emx1-Cre), Student’s t-test (duration of self-grooming), and Mann–Whitney U test (duration of digging). [file Image_1.TIF]
