# Supplementary material for: Shank3 Exons 14–16 Deletion in Glutamatergic Neurons Leads to Social and Repetitive Behavioral Deficits Associated With Increased Cortical Layer 2/3 Neuronal Excitability
Source: Front Cell Neurosci. 2019 Oct 10;13:458. doi: 10.3389/fncel.2019.00458 (PMC6795689; doi:10.3389/fncel.2019.00458)
Supplement: TABLE S1 — The order of behavior experiments. [file Data_Sheet_1.PDF]

Supplementary Table 1. The order of behavior experiments

This table shows the detailed order of behavior experiments of each lines.

1. The order of behavioral experiments for *Emx1-Cre;Shank3* $^{\Delta 14\text{-}16}$ mice								
Cohort1	Laboras test	Open-field test	Self-grooming	3-chamber teset	Elevated plus-maze	Light-dark test	Direct interaction	Adult USV
Cohort2	Open-field test	Self-grooming	Repetitive behavior	Elevated plus-maze	Light-dark test	3-chamber test	Direct interaction	Adult USV
Cohort3	Laboras test							
Cohort 4	Tube test							
2. The order of behavioral experiments for control *Emx1-Cre* mice								
Cohort1	Open-field	Repetitive behavior	Elevated plus-maze	Laboras test	Direct interaction			
Cohort2	Laboras test	Open-field	Elevated plus-maze	Repetitive behavior	Direct interaction			
