# Supplementary material for: Shank3 Exons 14–16 Deletion in Glutamatergic Neurons Leads to Social and Repetitive Behavioral Deficits Associated With Increased Cortical Layer 2/3 Neuronal Excitability
Source: Front Cell Neurosci. 2019 Oct 10;13:458. doi: 10.3389/fncel.2019.00458 (PMC6795689; doi:10.3389/fncel.2019.00458)
Supplement: TABLE S2 — Statistics table. [file Data_Sheet_2.PDF]

Supplementary Table 2. Statistics table

This table shows the details of statistical analyses and results

Figure		Assay Performed	Parameter (Unit)	Comparison					DescriptiveStatistics	Statistical Analysis					Normality test p values
				Variables 1	Variables 2	Age	n (animals)	Sex	Average ± SEM	Normality test	Statistical Test	Significance			
1	C	Western blot	Relative protein level (% WT)	Th	Shank3 a	12-13 weeks	5 pairs	Male & Female	1.38595 ± 0.612085		One sample t-test (Theoretical mean : 1.00)	t(4) = 0.630553	Two-tailed	P = 0.5626; ns	
					Shank3 c/d				1.25370 ± 0.316775			t(4) = 0.800868	Two-tailed	P = 0.4681; ns	
					Shank3 e				not detectable						
				Str	Shank3 a				1.06945 ± 0.280610			t(4) = 0.247493	Two-tailed	P = 0.8167; ns	
					Shank3 c/d				not detectable						
					Shank3 e				1.12863 ± 0.199988			t(4) = 0.643187	Two-tailed	P = 0.5551; ns	
				Hpc	Shank3 a				0.226216 ± 0.125780			t(4) = 6.15191	Two-tailed	P = 0.0035; **	
					Shank3 c/d				0.408352 ± 0.138753			t(4) = 4.26403	Two-tailed	P = 0.0130; *	
					Shank3 e				1.26910 ± 0.199625			t(4) = 1.34801	Two-tailed	P = 0.2489; ns	
				Ctx	Shank3 a				0.170008 ± 0.0469959			t(4) = 17.6610	Two-tailed	P < 0.0001; ***	
					Shank3 c/d				0.334670 ± 0.0381645			t(4) = 17.4332	Two-tailed	P < 0.0001; ***	
					Shank3 e				1.03597 ± 0.0764343			t(4) = 0.470575	Two-tailed	P = 0.6625; ns	
	E	Western blot	Relative protein level (% WT)	Shank1	Th	12-13 weeks	5 pairs	Male & Female	1.48694 ± 0.217459		One sample t-test (Theoretical mean : 1.00)	t(4) = 2.23924	Two-tailed	P = 0.0887; ns	
					Str				1.47952 ± 0.468001			t(4) = 1.02461	Two-tailed	P = 0.3635; ns	
					Hpc				1.11641 ± 0.140218			t(4) = 0.830194	Two-tailed	P = 0.4531; ns	
					Ctx				1.10306 ± 0.213121			t(4) = 0.483582	Two-tailed	P = 0.6540; ns	
				Shank2	Th				1.42513 ± 0.183838			t(4) = 2.3125	Two-tailed	P = 0.0818; ns	
					Str				1.14609 ± 0.108839			t(4) = 1.34227	Two-tailed	P = 0.2506; ns	
					Hpc				0.9162 ± 0.105842			t(4) = 0.791745	Two-tailed	P = 0.4728; ns	
					Ctx				1.10112 ± 0.177356			t(4) = 0.570135	Two-tailed	P = 0.5991; ns	
2	A	Neuornal Excitability (I-F curve)	Spike count	0	WT	P20-25	WT = 15 (4)<br>KO = 14 (3)	Male	0		Repeated measures of two-way ANOVA; Bonferroni's multiple comparison test	Interaction<br>F(11, 297) = 5.31921, P < 0.0001; ***<br>Genotype<br>F(1, 27) = 7.3568, P = 0.0115; *<br>Current<br>F(11, 297) = 375.165, P < 0.0001; ***		P > 0.9999; ns	
					KO				0						
				30	WT				0					P > 0.9999; ns	
					KO				0						
				60	WT				0					P > 0.9999; ns	
					WT				0						
				90	KO				0					P > 0.9999; ns	
					WT				0.571429						
				120	KO				0.333333					P > 0.9999; ns	
					WT				1.78571						
				150	WT				1.6					P > 0.9999; ns	
					KO				3.42857						
				1820	WT				3.06667					P > 0.9999; ns	
					KO				4.92857						
				210	WT				4.73333					P = 0.0485; *	
					KO				6.64286						
				240	WT				6.06667					P = 0.0072; **	
					KO				8.35714						
				270	WT				7.53333					P = 0.0088; **	
					WT				9.78571						
				300	KO				8.73333					P = 0.0120; *	
					WT				10.9286						
				330	KO				9.6					P = 0.0017; **	
					WT				12.1429						
	B	Neuornal Excitability (I-V curve)	Membrane potential (mV)	-150	WT	P20-25	WT = 15 (4)<br>KO = 14 (3)	Male	-90.84		Repeated measures of two-way ANOVA; Bonferroni's multiple comparison test	Interaction<br>F(16, 432) = 7.92821, P < 0.0001; ***<br>Genotype<br>F(1, 27) = 11.0699, P = 0.0025; **<br>Current<br>F(16, 432) = 1084.05, P < 0.0001; ***		P = 0.0001; ***	
					KO				-92.9286						
				-140	WT				-90.3					P = 0.0002; ***	
					KO				-92.3214						
				-130	WT				-89.7333					P = 0.0003; ***	
					KO				-91.7143						
				-120	WT				-89.16					P = 0.0005; ***	
					KO				-91.0786						
				-110	WT				-88.56					P = 0.0012; **	
					WT				-90.3929						
				-100	KO				-87.94					P = 0.0022; **	
					WT				-89.7						
				-90	KO				-87.3333					P = 0.0027; **	
					WT				-89.0714						
				-80	WT				-86.6667					P = 0.0073; **	
					KO				-88.2857						
				-70	WT				-85.8					P = 0.0205; *	
					KO				-87.2857						
				-60	WT				-85.1333					P = 0.0481; *	
					KO				-86.5						
				-50	WT				-84.4					P = 0.1655; ns	
					KO				-85.5714						
				-40	WT				-83.5333					P = 0.3313; ns	
					WT				-84.5714						
				-30	KO				-82.6667					P = 0.7008; ns	
					WT				-83.5						
				-20	KO				-81.8					P = 0.9571; ns	
					WT				-82.4286						
				-10	WT				-80.8667					P = 0.9965; ns	
					KO				-81.3571						
				0	WT				-79.8667					P > 0.9999; ns	
					KO				-80						
				10	WT				-78.7143					P > 0.9999; ns	
					KO				-78.9333						
	C	Neuornal Excitability (Input resistance)	Input Resistance (MΩ)	WT		P20-25	WT = 15 (4)<br>KO = 14 (3)	Male	93.052 ± 3.28304	O	Student's t-test	t(27) = 2.9198	Two-tailed	P = 0.0070; **	Yes; P = 0.5980; ns
				KO					114.906 ± 6.91001						Yes; P = 0.9353; ns
	D	Neuornal Excitability (I-F curve)	Spike count	0	WT	P20-24	WT = 18 (4)<br>KO = 17 (4)	Male	0		Repeated measures of two-way ANOVA; Bonferroni's multiple comparison test	Interaction<br>F(11, 363) = 4.40268, P < 0.0001; ***<br>Genotype<br>F(1, 33) = 7.0118, P = 0.0123; *<br>Current<br>F(11, 363) = 650.528, P < 0.0001; ***		P > 0.9999; ns	
					cKO				0						
				30	WT				0					P > 0.9999; ns	
					cKO				0						
				60	WT				0					P > 0.9999; ns	
					cKO				0						
				90	WT				0.166667					P = 0.9604; ns	
					cKO				0.882353						
				120	WT				1.61111					P = 0.4247; ns	
					cKO				2.82353						
				150	WT				3.27778					P = 0.0288; *	
					cKO				5.11765						
				1820	WT				5.11111					P = 0.0221; *	
					cKO				7						
				210	WT				6.88889					P = 0.0441; *	
					cKO				8.64706						
				240	WT				8.27778					P = 0.0076; **	
					cKO				10.3529						
				270	WT				9.88889					P = 0.0123; *	
					cKO				11.8824						
				300	WT				11.2778					P = 0.0528; ns	
					cKO				13						
				330	WT				12.4444					P = 0.0672; ns	
					cKO				14.1176						
	E	Neuornal Excitability (I-V curve)	Membrane potential (mV)	-150	WT	P20-24	WT = 18 (4)<br>KO = 17 (4)	Male	-92.6167		Repeated measures of two-way ANOVA; Bonferroni's multiple comparison test	Interaction<br>F(16, 528) = 10.9007, P < 0.0001; ***<br>Genotype<br>F(1, 33) = 11.8245, P = 0.0016; **<br>Current<br>F(16, 528) = 1533.74, P < 0.0001; ***		P < 0.0001; ***	
					cKO				-94.8765						
				-140	WT				-92.05					P < 0.0001; ***	
					cKO				-94.2176						
				-130	WT				-91.4667					P < 0.0001; ***	
					cKO				-93.5235						
				-120	WT				-90.8222					P = 0.0001; ***	
					cKO				-92.8059						
				-110	WT				-90.15					P = 0.0002; ***	
					cKO				-92.0529						
				-100	WT				-89.4611					P = 0.0007; ***	
					cKO				-91.2588						
				-90	WT				-88.6667					P = 0.0012; **	
					cKO				-90.4118						
				-80	WT				-87.8333					P = 0.0019; **	
					cKO				-89.5294						
				-70	WT				-87.1111					P = 0.0125; *	
					cKO				-88.5882						
				-60	WT				-86.2778					P = 0.1024; ns	
					cKO				-87.4706						
				-50	WT				-85.5					P = 0.3628; ns	
					cKO				-86.4706						
				-40	WT				-84.4444					P = 0.3684; ns	
					cKO				-85.4118						
				-30	WT				-83.3333					P = 0.8207; ns	
					cKO				-84.0588						
				-20	WT				-82.2222					P = 0.9830; ns	
					cKO				-82.7647						
				-10	WT				-81.1111					P > 0.9999; ns	
					cKO				-81.3529						
				0	WT				-80					P > 0.9999; ns	
					cKO				-79.9412						
				10	WT				-78.6667					P > 0.9999; ns	
					cKO				-78.3529						
	F	Neuornal Excitability (Input resistance)	Input Resistance (MΩ)	WT		P20-24	WT = 18 (4)<br>KO = 17 (4)	Male	113.447 ± 4.95231	O	Student's t-test	t(33) = 3.26129	Two-tailed	P = 0.0026; **	Yes; P = 0.7950; ns
				cKO					136.596 ± 5.0845						Yes; P = 0.3554; ns
3	A	mPFC mEPSCs	Frequency (Hz)	WT		P21-P26	WT = 17 (5)<br>KO = 17 (7)	Male	5.922059 ± 0.8175816	O	Student's t-test	t(32) = 2.806782	Two-tailed	P = 0.0084; **	Yes; P = 0.3256; ns
				KO					9.692157 ± 1.065727						Yes; P = 0.4952; ns
			Amplitude (pA)	WT					14.24987 ± 0.5349324	O	Student's t-test	t(32) = 1.501802	Two-tailed	p = 0.1430; ns	Yes; P = 0.2370; ns
				KO					15.3809 ± 0.5301208						Yes; P = 0.4085; ns
	B	mPFC mIPSCs	Frequency (Hz)	WT		P21-26	WT = 15 (5)<br>KO = 16 (6)	Male	18.49833 ± 0.9693636	O	Student's t-test	t(29) = 0.6892233	Two-tailed	P = 0.4962; ns	Yes; P = 0.4961; ns
				KO					19.68698 ± 1.40007						Yes; P = 0.4832; ns
			Amplitude (pA)	WT					29.20107 ± 1.442027	O	Student's t-test	t(29) = 0.5176422	Two-tailed	P = 0.6086; ns	Yes; P = 0.1164; ns
				KO					30.20509 ± 1.303763						Yes; P = 0.4049; ns
	C	mPFC mEPSCs	Frequency (Hz)	WT		P18-22	WT = 19 (4)<br>cKO = 16 (4)	Male	7.07807 ± 0.7484667	X	Mann-Whitney U test	U = 132.5000	Two-tailed	P = 0.5292; ns	No; P = 0.0212; *
				cKO					8.149479 ± 1.111042						Yes; P = 0.4244; ns
			Amplitude (pA)	WT					15.68456 ± 0.3855291	O	Student's t-test	t(33) = 1.889106	Two-tailed	P = 0.0677; ns	Yes; P = 0.1907; ns
				cKO					14.30079 ± 0.6549803						Yes; P = 0.6558; ns
	D	mPFC mIPSCs	Frequency (Hz)	WT		P18-22	WT = 14 (4)<br>cKO = 17 (4)	Male	6.597619 ± 0.7137043	O	Student's t-test	t(29) = 0.2852874	Two-tailed	P = 0.7775; ns	Yes; P = 0.2737; ns
				cKO					6.846078 ± 0.5291725						Yes; P = 0.6050; ns
			Amplitude (pA)	WT					40.3763 ± 2.077463	X	Mann-Whitney U test	U = 99.00000	Two-tailed	P = 0.4389; ns	No; P = 0.0307; *
				cKO					43.70187 ± 1.828974						Yes; P = 0.5011; ns
	E	mPFC sEPSCs	Frequency (Hz)	WT		P21-24	WT = 17 (3)<br>cKO = 17 (3)	Male	7.78039 ± 0.90194	O	Student's t-test	t(32) = 0.432438	Two-tailed	P = 0.6683; ns	Yes; P = 0.4115; ns
				KO					8.25392 ± 0.620952						Yes; P = 0.8977; ns
			Amplitude (pA)	WT					15.3974 ± 0.60918	O	Student's t-test	t(32) = 1.14353	Two-tailed	P = 0.2613; ns	Yes; P = 0.4210; ns
				KO					16.2936 ± 0.492969						Yes; P = 0.9429; ns
	F	mPFC sIPSCs	Frequency (Hz)	WT		P21-24	WT = 15 (3)<br>cKO = 18 (3)	Male	17.125 ± 0.712648	O	Student's t-test	t(31) =3.43473	Two-tailed	P = 0.0017; **	Yes; P = 0.3850; ns
				KO					23.0167 ± 1.44579						Yes; P = 0.8525; ns
			Amplitude (pA)	WT					31.8457 ± 1.59048	O	Student's t-test	t(31) = 0.652664	Two-tailed	P = 0.5188; ns	Yes; P = 0.2877; ns
				KO					33.1725 ± 1.29975						Yes; P = 0.9872; ns
	G	mPFC sEPSCs	Frequency (Hz)	WT		P20-25	WT = 15 (3)<br>cKO = 16 (3)	Male	7.08667 ± 0.754648	O	Student's t-test	t(29) = 0.516361	Two-tailed	P = 0.6095; ns	Yes; P = 0.2687; ns
				cKO					7.63438 ± 0.744452						Yes; P = 0.5360; ns
			Amplitude (pA)	WT					14.8745 ± 0.589684	O	Student's t-test	t(29) = 0.469201	Two-tailed	P = 0.6424; ns	Yes; P = 0.4288; ns
				cKO					14.5377 ± 0.421676						Yes; P = 0.0561; ns
			Frequency (Hz)	WT					6.93185 ± 0.929836	O	Student's t-test	t(36) = 0.768431	Two-tailed	P = 0.4472; ns	Yes; P = 0.3394; ns

	H	mPFC sIPSCs	Frequency (Hz)		[illegible]	P20-25	WT = 19 (3)<br>cKO = 19 (3)	Male	[illegible]	O	Student's t-test	t(36) = 0.768451	Two-tailed	P = 0.4472; ns	[illegible]
					cKO				6.03926 ± 0.696193						Yes; P = 0.2915; ns
			Amplitude (pA)		WT				39.0078 ± 2.06425	X	Mann-Whitney U test	U = 116.000	Two-tailed	P = 0.0612; ns	No; P = 0.0409; *
					cKO				44.4286 ± 1.51303						Yes; P = 0.2783; ns
4	A	Dorsolateral striatum mEPSCs	Frequency (Hz)		WT	P29-35	WT = 14 (3)<br>cKO = 17 (3)	Male	2.80476 ± 0.183763	O	Student's t-test	t(29) = 0.430988	Two-tailed	P = 0.6697; ns	Yes; P = 0.3707; ns
					cKO				2.97549 ± 0.325349						Yes; P = 0.3464; ns
			Amplitude (pA)		WT				19.2668 ± 0.850662	O	Student's t-test	t(29) = 0.336011	Two-tailed	P = 0.7393; ns	Yes; P = 0.3009; ns
					cKO				18.857 ± 0.856448						Yes; P = 0.6205; ns
	B	Dorsolateral striatum mIPSCs	Frequency (Hz)		WT	P34-41	WT = 15 (3)<br>cKO = 15 (3)	Male	6.14056 ± 0.547555	O	Student's t-test	t(28) = 0.4562	Two-tailed	P = 0.6518; ns	Yes; P = 0.9610; ns
					cKO				5.77278 ± 0.591709						Yes; P = 0.0689; ns
			Amplitude (pA)		WT				33.3456 ± 1.69674	O	Student's t-test	t(28) = 0.7202	Two-tailed	P = 0.4774; ns	Yes; P = 0.3179; ns
					cKO				35.2282 ± 1.98842						Yes; P = 0.5172; ns
5	A	Three-chamber S1-O	Time spent in sniffing (s)	WT	O	3 ~ 5 months	WT = 23<br>cKO = 20	Male	16.34353 ± 1.860471	O	Paired t-test	t(22) = 8.989818	Two-tailed	P < 0.0001; ***	Yes; P = 0.1665; ns
					S1				66.69138 ± 5.237831						Yes; P = 0.0847; ns
				cKO	O				23.57975 ± 2.725263	X	Wilcoxon signed rank test	W = -208.0000	Two-tailed	P = 0.0001; ***	Yes; P = 0.1218; ns
					S1				71.40659 ± 8.728646						No; P = 0.031; *
		Three-chamber S1-S2	Time spent in sniffing (s)	WT	S1				28.1609 ± 2.293138	O	Paired t-test	t(22) = 4.075990	Two-tailed	P = 0.0005; ***	Yes; P = 0.5275; ns
					S2				46.02766 ± 3.359488						Yes; P = 0.2800; ns
				cKO	S1				32.30831 ± 4.594513	O	Paired t-test	t(19) = 4.028673	Two-tailed	P = 0.0007; ***	Yes; P = 0.0746; ns
					S2				51.75735 ± 4.907646						Yes; P = 0.2290; ns
	B	Direct social interaction test	Male to male interaction time (s)	Nose to nose	WT	4 ~ 7months	WT = 12 pairs<br>cKO = 11 pairs	Male	11.87333 ± 0.6831437	O	Student's t-test	t(21) = 5.459407	Two-tailed	P < 0.0001; ***	Yes; P = 0.6571; ns
					cKO				20.56636 ± 1.48927						Yes; P = 0.2166; ns
				Following	WT				30.38167 ± 4.210473	O	Student's t-test	t(21) = 0.9938334	Two-tailed	P = 0.3316; ns	Yes; P = 0.2783; ns
					cKO				36.68455 ± 4.774339						Yes; P = 0.9508; ns
				Total interaction	WT				80.63 ± 6.446899	O	Student's t-test	t(21) = 3.499672	Two-tailed	P = 0.0021; **	Yes; P = 0.4123; ns
					cKO				119.1836 ± 9.115563						Yes; P = 0.9245; ns
	C	Tube test	Rank in tube test		WT	4 ~ 8months	WT = 11<br>cKO = 9	Male	2.54545 ± 0.365902	O	Student's t-test	t(18) = 0.190885	Two-tailed	P = 0.8508; ns	Yes; P = 0.2689; ns
					cKO				2.44444 ± 0.376796						Yes; P = 0.6600; ns
	D	Courtship USVs	Number of USV		WT	4 ~ 5 months	WT = 20<br>cKO = 20	Male	1302.5 ± 76.09729	O	Student's t-test	t(38) = 1.606599	Two-tailed	P = 0.1164; ns	Yes; P = 0.9119; ns
					cKO				1084.7 ± 112.1933						Yes; P = 0.411; ns
	E	Self-grooming test	Grooming duration (s)		WT	3 ~ 4 months	WT = 24<br>cKO = 21	Male	38.1621 ± 6.40562	X	Mann-Whitney U test	U = 185	Two-tailed	P = 0.1313; ns	No; P = 0.0113; *
					cKO				58.3119 ± 10.7013						No; P = 0.0136; *
	F	Repetitive behavior test	Duration (s)	Self-grooming	WT	3 ~ 5 months	WT = 16<br>cKO = 14	Male	16.65937 ± 3.557827	X	Mann-Whitney U test	U = 56.50000	Two-tailed	P = 0.0199; *	No; P = 0.0438; *
					cKO				30.18571 ± 4.420166						Yes; P = 0.8449; ns
				Digging	WT				15.68437 ± 1.646712	O	Student's t-test	t(28) = 0.4359615	Two-tailed	P = 0.6662; ns	Yes; P = 0.4021; ns
					cKO				17.21786 ± 3.261415						Yes; P = 0.2613; ns
	G	Laboras	Time spent in self-grooming (s)	2	WT	2 ~ 4 months	WT = 13<br>cKO = 14	Male	879.6639		Repeated measures of two-way ANOVA	Interaction<br>F(47, 1175) = 1.051735, P = 0.3798 ; ns<br><br>Genotype<br>F(1, 25) = 0.7510219, P = 0.3944; ns<br><br>Time<br>F(47, 1175) = 30.97872, P < 0.0001; ***			
					cKO				911.14						
				4	WT				851.3955						
					cKO				855.9415						
				6	WT				817.5384						
					cKO				831.8821						
				8	WT				752.713						
					cKO				956.1622						
				10	WT				703.3369						
					cKO				857.98						
				12	WT				876.1139						
					cKO				947.0571						
				14	WT				507.2408						
					cKO				487.1586						
				16	WT				376.0838						
					cKO				311.7043						
				18	WT				403.2954						
					cKO				373.6557						
				20	WT				351.6639						
					cKO				317.35						
				22	WT				504.6554						
					cKO				663.1935						
				24	WT				783.7169						
					cKO				775.2401						
				26	WT				1200.158						
					cKO				1066.995						
				28	WT				859.6261						
					cKO				1116.344						
				30	WT				784.9431						
					cKO				984.7357						
				32	WT				839.5508						
					cKO				774.835						
				34	WT				748.6223						
					cKO				753.6957						
				36	WT				721.2255						
					cKO				750.8057						
				38	WT				355.8069						
					cKO				278.1586						
				40	WT				337.6262						
					cKO				244.8536						
				42	WT				479.6053						
					cKO				386.245						
				44	WT				351.6862						
					cKO				443.575						
				46	WT				633.3378						
					cKO				568.0479						
				48	WT				488.5131						
					cKO				511.0178						
				50	WT				1146.482						
					cKO				1178.867						
				52	WT				1043.437						
					cKO				1062.341						
				54	WT				750.42						
					cKO				893.8071						
				56	WT				952.8446						
					cKO				974.8028						
				58	WT				857.6693						
					cKO				889.0172						
				60	WT				710.3062						
					cKO				777.785						
				62	WT				431.3277						
					cKO				283.6721						
				64	WT				302.04						
					cKO				424.1093						
				66	WT				337.8077						
					cKO				287.0279						
				68	WT				356.6131						
					cKO				364.4929						
				70	WT				563.4778						
					cKO				529.3235						
				72	WT				531.723						
					cKO				620.3943						
				74	WT				1155.118						
					cKO				1410.724						
				76	WT				1063.229						
					cKO				1113.769						
				78	WT				763.6684						
					cKO				990.7993						
				80	WT				779.1193						
					cKO				1037.792						
				82	WT				892.1024						
					cKO				911.0006						
				84	WT				815.9662						
					cKO				861.9993						
				86	WT				334.25						
					cKO				441.3315						
				88	WT				355.2085						
					cKO				364.9929						
				90	WT				291.0284						
					cKO				367.8671						
				92	WT				350.0392						
					cKO				369.6772						
				94	WT				434.1653						
					cKO				552.88						
				96	WT				721.7946						
					cKO				722.335						
	H	Laboras	Time spent in repetitive behavior (s)	Climbing	WT	2 ~ 4 months	WT = 13<br>cKO = 14	Male	19388.91 ± 1762.47	O	Student's t-test	t(25) = 2.034558	Two-tailed	P = 0.0526; ns	Yes; P = 0.9922; ns
					cKO				14799.63 ± 1431.285						Yes; P = 0.9931; ns
				Rearing	WT				3211.546 ± 363.0022	O	Student's t-test	t(25) = 0.5563334	Two-tailed	P = 0.5829; ns	Yes; P = 0.1651; ns
					cKO				3428.289 ± 166.1078						Yes; P = 0.6791; ns
				Grooming	WT				20965.25 ± 1229.023	O	Student's t-test	t(25) = 1.152846	Two-tailed	P = 0.2599; ns	Yes; P = 0.4189; ns
					cKO				22910.28 ± 1157.982						Yes; P = 0.4372; ns
				Drinking	WT				1059.81 ± 103.1749	X	Mann-Whitney U test	U = 62	Two-tailed	P = 0.1667; ns	Yes; P = 0.4087; ns
					cKO				1183.268 ± 70.98996						No; P = 0.036; *
				Eating	WT				7417.233 ± 664.7468	O	Student's t-test	t(25) = 1.952250	Two-tailed	P = 0.0622; ns	Yes; P = 0.4266; ns
					cKO				8849.496 ± 345.8435						Yes; P = 0.4192; ns
	A	Open-field test	Distance moved (m)	10	WT	3 ~ 4 months	WT = 23<br>cKO = 21	Male	39.94807		Repeated measures of two-way ANOVA	Interaction<br>F(5, 210) = 1.143107, P = 0.3387; ns<br><br>Genotype<br>F(1, 42) =0.1625639, P = 0.6889; ns<br><br>Time<br>F(5, 210) = 218.8782, P < 0.0001; ***			
					cKO				40.50136						
				20	WT				28.67298						
					cKO				29.67041						
				30	WT				26.68014						
					cKO				25.13712						
				40	WT				23.17891						
					cKO				21.60225						
				50	WT				20.59448						
					cKO				20.44867						
				60	WT				18.10495						
					cKO				16.50636						
			Total distance moved (m)		WT				157.1813 ± 5.878035	O	Student's t-test	t(42) = 0.4031584	Two-tailed	P = 0.6889; ns	Yes; P = 0.7260; ns
					cKO				153.8682 ± 5.701412						Yes; P = 0.2868; ns
			Time in center (%)		WT				21.11794 ± 1.599615	O	Student's t-test	t(42) = 0.3422001	Two-tailed	P = 0.7339; ns	Yes; P = 0.5308; ns
					cKO				20.35159 ± 1.556507						Yes; P = 0.7325; ns
				2	WT				31.23146						
					cKO				34.29186						
				4	WT				27.25654						
					cKO				23.78372						
				6	WT				21.41515						
					cKO				19.15543						
				8	WT				9.914539						
					cKO				14.81422						
				10	WT				6.300538						
					cKO				10.60379						
				12	WT				14.82977						
					cKO				19.962						
				14	WT				10.73492						

6	B	Laboras	Distance moved (m)	14	cKO	2 ~ 4 months	WT = 13<br>cKO = 14	Male	12.15714		Repeated measures of two-way ANOVA	Interaction<br>F(47, 1175) =0.7374643, P = 0.9059 ; ns<br><br>Genotype<br>F(1, 25) = 1.108328, P = 0.3025; ns<br><br>Time<br>F(47, 1175) =48.1637, P < 0.0001; ***			
				16	WT				3.044077						
					cKO				2.844429						
				18	WT				2.962923						
					cKO				3.860857						
				20	WT				2.607769						
					cKO				3.508571						
				22	WT				3.039385						
					cKO				4.220714						
				24	WT				4.087923						
					cKO				5.153714						
				26	WT				13.484						
					cKO				15.79286						
				28	WT				26.61231						
					cKO				27.70157						
				30	WT				20.01392						
					cKO				24.71143						
				32	WT				9.522						
					cKO				12.5465						
				34	WT				11.19431						
					cKO				9.523929						
				36	WT				13.86592						
					cKO				17.6545						
				38	WT				3.835538						
					cKO				6.037071						
				40	WT				2.280692						
					cKO				2.757357						
				42	WT				2.439						
					cKO				3.736428						
				44	WT				3.073						
					cKO				4.681214						
				46	WT				4.579308						
					cKO				5.072857						
				48	WT				4.299462						
					cKO				5.137786						
				50	WT				15.57092						
					cKO				16.7595						
				52	WT				17.93492						
					cKO				19.15893						
				54	WT				13.37962						
					cKO				15.75893						
				56	WT				11.32285						
					cKO				13.55407						
				58	WT				7.171154						
					cKO				12.03486						
				60	WT				14.40838						
					cKO				15.55729						
				62	WT				3.368923						
					cKO				4.340428						
				64	WT				2.593						
					cKO				4.991928						
				66	WT				2.101154						
					cKO				3.003929						
				68	WT				2.959923						
					cKO				3.586						
				70	WT				3.955153						
					cKO				5.230643						
				72	WT				4.998461						
					cKO				6.419929						
				74	WT				19.467						
					cKO				18.15307						
				76	WT				16.97577						
					cKO				15.02593						
				78	WT				14.204						
					cKO				15.48586						
				80	WT				12.00585						
					cKO				11.432						
				82	WT				12.06431						
					cKO				11.43214						
				84	WT				14.36631						
					cKO				15.07671						
				86	WT				3.266077						
					cKO				5.4265						
				88	WT				3.68677						
					cKO				4.701357						
				90	WT				3.275307						
					cKO				3.642143						
				92	WT				3.047923						
					cKO				3.496643						
				94	WT				3.877692						
					cKO				3.155857						
				96	WT				3.973769						
					cKO				6.720429						
			Total distance moved (m)	First 6hrs	WT				72.40985 ± 7.219538	O	Student's t-test	t(25) = 0.4441215	Two-tailed	0.6608; ns	Yes; P = 0.2634; ns
					cKO				77.231 ± 8.027246						Yes; P = 0.1139; ns
				Last 72hrs	WT				395.8998 ± 89.82525	X	Mann-Whitney U test	U = 78.00000	Two-tailed	0.5441; ns	No; P < 0.0001; ***
					cKO				369.4986 ± 28.1803						Yes; P = 0.2645; ns
	C	Elevated plus-maze test	Time in arms (s)	WT	Open	3 ~ 5 months	WT = 23<br>cKO = 20	Male	73.79107 ± 9.407399	O	Paired t-test	t(22) = 12.20917	Two-tailed	P < 0.0001; ***	Yes; P = 0.5507; ns
					Closed				308.7572 ± 10.89522						Yes; P = 0.3002; ns
				KO	Open				110.3346 ± 9.881269	O	Paired t-test	t(19) = 7.029055	Two-tailed	P < 0.0001; ***	Yes; P = 0.7168; ns
					Closed				268.5162 ± 13.95163						Yes; P = 0.7261; ns
			Time in open arms (%)	WT					15.37314 ± 1.959875	O	Student's t-test	t(41) = 2.674541	Two-tailed	P = 0.0107; *	Yes; P = 0.5507; ns
				cKO					22.98639 ± 2.058598						Yes; P = 0.7168; ns
	D	Light-dark test	Time in light chamber (s)	WT		3 ~ 5 months	WT = 23<br>cKO = 21	Male	34.45403 ± 2.246006	O	Student's t-test	t(42) = 0.1065583	Two-tailed	P = 0.9156; ns	Yes; P = 0.1391; ns
				cKO					34.80816 ± 2.459013						Yes; P = 0.5149; ns
			Number of transition (s)	WT					32.69565 ± 1.941267	O	Student's t-test	t(42) = 0.7669833	Two-tailed	P = 0.4474; ns	Yes; P = 0.9912; ns
				cKO					34.80952 ± 1.948894						Yes; P = 0.5388; ns
Suppl 1	A	Laboras	Distance moved (m)	2	WT	3 months	WT = 19<br>Emx-Cre1 = 15	Male	46.61263		Repeated measures of two-way ANOVA	Interaction<br>F(47, 1504) = 0.5220582, P = 0.997 ; ns<br><br>Genotype<br>F(1, 32) = 0.862411, P = 0.3600; ns<br><br>Time<br>F(47, 1504) = 92.2774, P < 0.0001; ***			
					Emx1-Cre				45.69347						
				4	WT				34.28221						
					Emx1-Cre				34.45347						
				6	WT				31.68589						
					Emx1-Cre				27.10113						
				8	WT				18.97095						
					Emx1-Cre				17.00607						
				10	WT				11.371						
					Emx1-Cre				10.30867						
				12	WT				14.4099						
					Emx1-Cre				11.24647						
				14	WT				6.312053						
					Emx1-Cre				6.096733						
				16	WT				2.802316						
					Emx1-Cre				3.8366						
				18	WT				2.063473						
					Emx1-Cre				2.158267						
				20	WT				1.612368						
					Emx1-Cre				2.730733						
				22	WT				2.39						
					Emx1-Cre				3.225133						
				24	WT				2.757842						
					Emx1-Cre				2.867733						
				26	WT				23.84695						
					Emx1-Cre				22.55906						
				28	WT				30.81021						
					Emx1-Cre				28.54553						
				30	WT				20.45347						
					Emx1-Cre				20.85607						
				32	WT				16.31832						
					Emx1-Cre				11.81133						
				34	WT				12.19795						
					Emx1-Cre				11.74333						
				36	WT				14.59274						
					Emx1-Cre				11.84307						
				38	WT				5.088105						
					Emx1-Cre				4.5814						
				40	WT				3.920737						
					Emx1-Cre				4.369933						
				42	WT				2.101789						
					Emx1-Cre				3.2524						
				44	WT				1.997631						
					Emx1-Cre				2.278667						
				46	WT				3.901685						
					Emx1-Cre				3.298733						
				48	WT				3.45979						
					Emx1-Cre				4.0268						
				50	WT				24.74668						
					Emx1-Cre				21.282						
				52	WT				23.55895						
					Emx1-Cre				19.07553						
				54	WT				17.75489						
					Emx1-Cre				16.54407						
				56	WT				13.62453						
					Emx1-Cre				11.9566						
				58	WT				8.544789						
					Emx1-Cre				8.7828						
				60	WT				11.70695						
					Emx1-Cre				11.1226						
				62	WT				5.212579						
					Emx1-Cre				4.826533						
				64	WT				3.258474						
					Emx1-Cre				2.853867						
				66	WT				2.662632						
					Emx1-Cre				2.954267						
				68	WT				3.237421						
					Emx1-Cre				3.146067						
				70	WT				3.170684						
					Emx1-Cre				3.500933						
				72	WT				3.988						
					Emx1-Cre				3.850934						
				74	WT				23.657						
					Emx1-Cre				21.355						

				76	WT				17.93616						
					Emx1-Cre				16.8676						
				78	WT				16.7879						
					Emx1-Cre				14.62233						
				80	WT				16.70253						
					Emx1-Cre				15.51553						
				82	WT				11.75147						
					Emx1-Cre				9.902932						
				84	WT				14.00726						
					Emx1-Cre				10.81667						
				86	WT				3.344474						
					Emx1-Cre				3.524333						
				88	WT				3.855474						
					Emx1-Cre				3.452						
				90	WT				2.534158						
					Emx1-Cre				2.267933						
				92	WT				2.275632						
					Emx1-Cre				3.088467						
				94	WT				3.075842						
					Emx1-Cre				3.2704						
				96	WT				3.899158						
					Emx1-Cre				3.622667						
			Total distance moved (m)	First 6hrs	WT				112.5807 ± 8.19171	X	Mann-Whitney U test	U = 131.0000	Two-tailed	P = 0.7028; ns	No; P = 0.0270; *
					Emx1-Cre				107.2481 ± 8.281446						Yes; P = 0.6818; ns
				Last 72hrs	WT				379.983 ± 26.13214	O	Student's t-test	t(32) = 0.9789865	Two-tailed	0.3349; ns	Yes; P = 0.0963; ns
					Emx1-Cre				347.3684 ± 17.47886						Yes; P = 0.3453; ns
			Time spent in repetitive behavior (s)	Climbing	WT				19699.38 ± 1738.119	O	Student's t-test	t(32) = 0.3819945	Two-tailed	P = 0.7050; ns	Yes; P = 0.7066; ns
					Emx1-Cre				18626.21 ± 2271.87						Yes; P = 0.3660; ns
				Rearing	WT				4303.871 ± 299.7794	O	Student's t-test	t(32) = 0.9857117	Two-tailed	P = 0.3317; ns	Yes; P = 0.3329; ns
					Emx1-Cre				3936.083 ± 177.3922						Yes; P = 0.9328; ns
				Grooming	WT				16534.45 ± 667.7766	X	Mann-Whitney U test	U = 137.0000	Two-tailed	P = 0.8623; ns	Yes; P = 0.5211; ns
					Emx1-Cre				16958.91 ± 827.8669						No; P = 0.0133; *
				Driking	WT				1090.059 ± 111.4208	O	Student's t-test	t(32) = 0.5464461	Two-tailed	P = 0.5885; ns	Yes; P = 0.1556; ns
					Emx1-Cre				1006.339 ± 98.8153						Yes; P = 0.5864; ns
				Eating	WT				7136.417 ± 373.3758	O	Student's t-test	t(32) = 0.5056184	Two-tailed	P = 0.6166; ns	Yes; P = 0.8150; ns
					Emx1-Cre				7444.175 ± 496.1736						Yes; P = 0.5938; ns
	B	Open-field test	Distance moved (m)	10	WT	2 ~ 3 months	WT = 19<br>Emx-Cre1 = 16	Male	38.61364		Repeated measures of two-way ANOVA	Interaction<br>F(5, 165) =0.32838, P = 0.8953; ns<br><br>Genotype<br>F(1, 33) = 0.000670295, P = 0.9795; ns<br><br>Time<br>F(5, 165) =195.8919, P < 0.0001; ***			
					Emx1-Cre				39.33936						
				20	WT				28.44697						
					Emx1-Cre				28.48389						
				30	WT				22.46642						
					Emx1-Cre				21.63589						
				40	WT				14.49395						
					Emx1-Cre				14.0702						
				50	WT				10.75433						
					Emx1-Cre				9.997362						
				60	WT				10.6182						
					Emx1-Cre				12.12469						
			Total distance moved (m)	WT					125.3952 ± 7.536047	O	Student's t-test	t(33) =0.02595042	Two-tailed	P = 0.9795; ns	Yes; P = 0.9107; ns
				Emx1-Cre					125.6537 ± 6.128843						Yes; P = 0.6176; ns
			Time in center (%)	WT					12.99106 ± 1.223812	O	Student's t-test	t(33) = 0.4432647	Two-tailed	P = 0.6605; ns	Yes; P = 0.1926; ns
				Emx1-Cre					12.13639 ± 1.518466						Yes; P = 0.3790; ns
	C	Elevated plus-maze test	Time in arms (s)	WT	Open	3 months	WT = 18<br>Emx-Cre1 = 16	Male	67.78233 ± 9.409456	O	Paired t-test	t(17) = 12.38622	Two-tailed	P < 0.0001; ***	Yes; P = 0.4776; ns
					Closed				302.4245 ± 10.34226						Yes; P = 0.6244; ns
				Emx1-Cre	Open				57.1562 ± 6.239703	O	Paired t-test	t(15) = 18.04467	Two-tailed	P < 0.0001; ***	Yes; P = 0.3145; ns
					Closed				308.9554 ± 9.347855						Yes; P = 0.5378; ns
			Time in open arms (%)	WT					14.12132 ± 1.960303	O	Student's t-test	t(32) = 0.9165099	Two-tailed	P = 0.3663; ns	Yes; P = 0.4776; ns
				Emx1-Cre					11.90754 ± 1.299938						Yes; P = 0.3145; ns
	D	Direct social interaction test	Male to male interaction time (s)	Nose to nose	WT	5 ~ 7 months	WT = 7 pairs<br>Emx-Cre1 = 5 pairs	Male	15.25286 ± 0.6416115	X	Mann-Whitney U test	U = 6.000000	Two-tailed	P = 0.0732; ns	N too small to pass the normality test
					Emx1-Cre				25.764 ± 6.297169						
				Following	WT				23.07143 ± 3.363818	X	Mann-Whitney U test	U = 17.00000	Two-tailed	P = 1.000; ns	
					Emx1-Cre				22.85 ± 3.358772						
				Total interaction	WT				58.44143 ± 4.97347	X	Mann-Whitney U test	U = 9.000000	Two-tailed	P = 0.2020; ns	
					Emx1-Cre				71.82 ± 8.433488						
	E	Repetitive behavior test	Duration (s)	Self-grooming	WT	2 ~ 4 months	WT = 19<br>Emx-Cre1 = 16	Male	18.44737 ± 2.100226	O	Student's t-test	t(33) = 1.953024	Two-tailed	P = 0.0593; ns	Yes; P = 0.6292; ns
					Emx1-Cre				28.09687 ± 4.782125						Yes; P = 0.4828; ns
				Digging	WT				68.75158 ± 8.475587	X	Mann-Whitney U test	U = 132.0000	Two-tailed	P = 0.5185; ns	Yes; P = 0.0556; ns
					Emx1-Cre				57.31 ± 7.604483						No; P = 0.0321; *
